# Small molecule inhibitors of α-synuclein oligomers identified by targeting early dopamine-mediated motor impairment in *C. elegans*

**DOI:** 10.1186/s13024-021-00497-6

**Published:** 2021-11-12

**Authors:** Kevin S. Chen, Krystal Menezes, Jarlath B. Rodgers, Darren M. O’Hara, Nhat Tran, Kazuko Fujisawa, Seiya Ishikura, Shahin Khodaei, Hien Chau, Anna Cranston, Minesh Kapadia, Grishma Pawar, Susan Ping, Aldis Krizus, Alix Lacoste, Scott Spangler, Naomi P. Visanji, Connie Marras, Nour K. Majbour, Omar M. A. El-Agnaf, Andres M. Lozano, Joseph Culotti, Satoshi Suo, William S. Ryu, Suneil K. Kalia, Lorraine V. Kalia

**Affiliations:** 1 Krembil Brain Institute, Toronto Western Hospital, University Health Network, Toronto, ON Canada; 2 Donnelly Centre, University of Toronto, Toronto, ON Canada; 3 BenevolentAI, New York, NY USA; 4 IBM Research-Almaden, San Jose, CA USA; 5 Edmond J. Safra Program in Parkinson’s Disease and the Morton and Gloria Shulman Movement Disorders Clinic, Division of Neurology, Department of Medicine, Toronto Western Hospital, University Health Network, Toronto, ON Canada; 6 Division of Neurology, Department of Medicine, University of Toronto, Toronto, ON Canada; 7 Neurological Disorders Research Center, Qatar Biomedical Research Institute (QBRI), Hamad Bin Khalifa University (HBKU), Qatar Foundation, Doha, Qatar; 8 Division of Neurosurgery, Department of Surgery, University of Toronto, Toronto, ON Canada; 9 Lunenfeld-Tanenbaum Research Institute, Mount Sinai Hospital, Toronto, ON Canada; 10 Department of Pharmacology, Faculty of Medicine, Saitama Medical University, Saitama, Japan; 11 Department of Physics, University of Toronto, Toronto, ON Canada; 12 KITE and CRANIA, University Health Network, Toronto, ON Canada; 13 Tanz Centre for Research in Neurodegenerative Diseases, University of Toronto, Toronto, ON Canada

**Keywords:** Alpha-synuclein, Animal model, Artificial intelligence, Drug discovery, Machine learning, Natural language processing, Neurodegeneration, Oligomers, Parkinson’s disease

## Abstract

**Background:**

Parkinson’s disease is a disabling neurodegenerative movement disorder characterized by dopaminergic neuron loss induced by α-synuclein oligomers. There is an urgent need for disease-modifying therapies for Parkinson’s disease, but drug discovery is challenged by lack of in vivo models that recapitulate early stages of neurodegeneration. Invertebrate organisms, such as the nematode worm *Caenorhabditis elegans*, provide in vivo models of human disease processes that can be instrumental for initial pharmacological studies.

**Methods:**

To identify early motor impairment of animals expressing α-synuclein in dopaminergic neurons, we first used a custom-built tracking microscope that captures locomotion of single *C. elegans* with high spatial and temporal resolution. Next, we devised a method for semi-automated and blinded quantification of motor impairment for a population of simultaneously recorded animals with multi-worm tracking and custom image processing. We then used genetic and pharmacological methods to define the features of early motor dysfunction of α-synuclein-expressing *C. elegans*. Finally, we applied the *C. elegans* model to a drug repurposing screen by combining it with an artificial intelligence platform and cell culture system to identify small molecules that inhibit α-synuclein oligomers. Screen hits were validated using in vitro and in vivo mammalian models.

**Results:**

We found a previously undescribed motor phenotype in transgenic α-synuclein *C. elegans* that correlates with mutant or wild-type α-synuclein protein levels and results from dopaminergic neuron dysfunction, but precedes neuronal loss. Together with artificial intelligence-driven in silico and in vitro screening, this *C. elegans* model identified five compounds that reduced motor dysfunction induced by α-synuclein. Three of these compounds also decreased α-synuclein oligomers in mammalian neurons, including rifabutin which has not been previously investigated for Parkinson’s disease. We found that treatment with rifabutin reduced nigrostriatal dopaminergic neurodegeneration due to α-synuclein in a rat model.

**Conclusions:**

We identified a *C. elegans* locomotor abnormality due to dopaminergic neuron dysfunction that models early α-synuclein-mediated neurodegeneration. Our innovative approach applying this in vivo model to a multi-step drug repurposing screen, with artificial intelligence-driven in silico and in vitro methods, resulted in the discovery of at least one drug that may be repurposed as a disease-modifying therapy for Parkinson’s disease.

**Supplementary Information:**

The online version contains supplementary material available at 10.1186/s13024-021-00497-6.

## Background

Parkinson’s disease (PD) is the most common neurodegenerative movement disorder, and its prevalence is projected to double over the next two decades [1]. Hence, there is an urgent need for the discovery of disease-modifying therapies that will slow the neurodegenerative process and thereby reduce morbidity. PD is defined by motor impairment due to selective degeneration of dopaminergic neurons in the substantia nigra pars compacta (SN). Abnormal accumulation of the protein α-synuclein and its aggregation into oligomers and fibril-containing inclusions, termed Lewy bodies, are also defining features. Missense mutations in *SNCA*, the gene encoding α-synuclein, promote aggregation of mutated α-synuclein and cause inherited monogenic forms of PD (e.g., A30P or A53T mutation). In addition, increased expression of non-mutated α-synuclein due to multiplications of *SNCA* or small nucleotide polymorphisms in *SNCA* leads to PD, providing strong evidence that α-synuclein can mediate neurodegeneration in both its mutant and wild-type forms [2]. Thus, α-synuclein has emerged as a promising therapeutic target for disease modification in PD [3].

To date, human trials aimed at discovering disease-modifying therapies for PD have been unsuccessful [4]. An important lesson from these failed attempts is that therapies may need to be initiated early in the neurodegenerative process, when interventions are expected to have their greatest impact [5]. Yet, ongoing preclinical efforts still rely on animal models of later disease stages, often when over 50% of dopaminergic neurons are already lost. Hence, there is a need for in vivo models that recapitulate early stages of neurodegeneration for PD drug discovery. Invertebrate organisms, such as the nematode worm *Caenorhabditis elegans*, are instrumental for in vivo modelling of human disease processes to test potential pharmacological treatments [6]. *C. elegans* is particularly amenable to modelling aspects of neurological diseases, such as PD, because the animal has a well-characterized nervous system, which uses many of the same neurotransmitters found in humans (e.g., dopamine) and mediates a diversity of motor behaviours.

Transgenic *C. elegans* strains expressing α-synuclein have been developed but none provide a sensitive indicator of the earlier stages of dopaminergic neuron degeneration [7]. In one of the commonly used models, wild-type α-synuclein fused to a fluorescent protein is expressed only within body wall muscle cells where large intramuscular protein inclusions of α-synuclein form spontaneously [8]. While this model can be useful to examine α-synuclein aggregation, it has limited relevance to PD since intramuscular α-synuclein inclusions do not occur in the disease. Another commonly used transgenic *C. elegans* co-expresses wild-type or mutant α-synuclein with a fluorescent protein in dopaminergic neurons. Neurodegeneration is observed by changes in morphology of the fluorescent neurons, including shortened neuritic processes and rounding of soma, as well as overt neuron loss [9, 10]. However, these structural changes reflect severely compromised neurons and represent dopaminergic neuron degeneration at advanced stages.

Here we identify an early motor phenotype of transgenic *C. elegans* expressing α-synuclein in dopaminergic neurons. We demonstrate that this motor abnormality is due to α-synuclein-mediated dopaminergic neuron dysfunction and occurs prior to neuronal loss. We apply this in vivo model downstream of a combined artificial intelligence (AI)-driven in silico and in vitro drug screen to identify compounds that reduce the *C. elegans* motor impairment. Further, we validate the utility of this approach by demonstrating that one of our top hits lowers pathophysiological conformations of α-synuclein and mitigates the loss of dopaminergic neurons in a rat model of PD. Thus, we provide an innovative and validated system for the discovery of potential disease-modifying drugs for PD.

## Methods

### *C. elegans* strains

*C. elegans* were maintained at room temperature (21 °C) on nematode growth medium (NGM) agar plates with *E. coli* OP50 as a food source as previously described [11]. The N2 (or Bristol) strain is the canonical wild-type strain used in most *C. elegans* research and was obtained from Caenorhabditis Genetics Center (CGC; University of Minnesota, St. Paul, MN, USA). The following additional strains were obtained from CGC: BR2823 (*by155*), BZ555 (*egIs1*[*dat-1p::gfp*]), CB102 (*e102*), CB587 (*e587*), CB933 (*e245*), and CB1112 (*e1112*). Strains containing the following transgenes were obtained from National BioResource Project (NBRP; Japan): *tmIs903*, *tmIs904*, *tmIs905*, *tmIs1082*, *tmIs1083*, and *tmIs1084*. VN305, VN306, and VN307 were obtained by crossing with YT2022 (*tzIs3*[*cre::gfp*]) [12] (kindly provided by Dr. Yoshishige Kimura, Kanagawa University of Human Services) with wild-type α-synuclein, A30P α-synuclein, and A53T α-synuclein transgenic animals, respectively (kindly provided by Dr. Takeshi Iwatsubo, University of Tokyo) [9]. VN310 was obtained by backcrossing YT2022 with N2. VN162 was obtained by crossing YT2022 with CB1112. Control GFP animals were obtained by crossing VN310 with BZ555 and used as a control for ectopic protein expression in dopaminergic neurons, since GFP was expressed under the same *dat-1* promoter as α-synuclein. VN306 was crossed with BR2823 to produce double mutants. VN306 was crossed with BZ555 to use GFP to track dopaminergic neuron loss over time.

### *C. elegans* locomotion

Animals were synchronized by isolating eggs using alkaline hypochlorite treatment and hatching overnight. Larvae were plated onto NGM agar plates with OP50 and grown for 4 days before recording locomotion. For individual *C. elegans* recordings, a single adult animal was randomly picked, washed for 1 to 2 min in M9 buffer, and then transferred to a fresh NGM plate without food for imaging. Video images were acquired (2 frames per second) for up to 6 min using a sCMOS camera (pco.edge, PCO) mounted on a stereomicroscope (Olympus MVX10 MacroView) controlled using the μManager software [13]. A total of 40 individual animals were imaged for each strain. For *C. elegans* population recordings, ten adult animals were randomly removed using an eyelash pick, washed for 1 min in M9 buffer, and then transferred to a fresh NGM plate without food, with a ring of copper sulfate solution (150 mM) around the area of recording (a chemorepellent to keep the animals in the recording frame). Exploratory locomotion was recorded using a CCD camera (GX1920, Allied Vision) which was attached to a dissecting stereomicroscope (Leica M165C, 1.0x PlanApo lens). A Labview program captured video (2 frames per second or 10 frames per second) at 1936 × 1456 resolution. Using ImageJ, the displacement, perimeter, and area of each animal was calculated, from which a circularity value was computed using the following formula: circularity = 4π(A/P^2^), where A is the area of the animal’s body and P is the perimeter of the animal’s body (both expressed in arbitrary pixel units). We set a cut-off for coiling at a circularity value of 0.6; an animal that had a circularity value > 0.6 was considered to be coiling. Using MATLAB (Mathworks), we calculated various behavioural metrics including the coiler score, which is defined as the percentage of frames with a circularity value > 0.6. Speed was automatically computed from the ImageJ based WrmTrk plugin as the distance travelled by each animal (in pixels) over the amount of time that it was tracked (in seconds) [14]. We determined average speed by calculating the weighted average of each tracked animal’s speed within a single population of animals. On the day of the recording for dopamine or raclopride treatment, adult animals were washed for 1 min in M9 buffer, treated in a solution containing either drug for 1 min, and then placed onto fresh NGM plates without food, with a copper sulfate solution ring around the area of recording. For all other drug treatment experiments, larvae were first grown for 2 days after hatching and then treated on drug plates for 3 days prior to recording, since drug treatment of younger larvae resulted in abnormal development and unhealthy animals. Drug plates were prepared by adding each drug to LB medium with OP50 before spreading onto fresh NGM plates. The solvent used to dissolve each drug was used as vehicle control for the drug treatment experiments (M9 buffer for dopamine, distilled water for raclopride, DMSO for all other drugs). A concentration of 100 μM was used for treatment with rapamycin based on previous reports [15] and the same concentration was used for treatment with acetaminophen, caffeine, losartan, and rifabutin.

### *C. elegans* lifespan

Synchronized larvae were plated onto NGM plates with food and grown for 2 days. Thirty L4 animals were picked and transferred to a fresh NGM plate containing floxuridine (FUDR; 0.005 mg/mL) with food (day 0). A total of 15 plates were prepared for each *C. elegans* strain (total *N* = 450 animals per strain). Animals were scored as alive or dead every 2 days until no alive animals remained on the plate. An animal was marked as dead and removed from the plate if it no longer exhibited movement and did not respond to prodding with an eyelash pick. Censored animals included those *C. elegans* that could not be found (e.g., burrowed into the agar) or died due to desiccation on the walls of the plate. An individual animal’s lifespan was the age at which it was scored as dead, and mean lifespan was calculated for each plate. Mean lifespan for each *C. elegans* strain was calculated as the average of the 15 plates.

### Enzyme-linked immunosorbent assay (ELISA)

Synchronized larvae were plated onto NGM plates with food and grown for 4 days. Animals were collected, washed, and pelleted in M9 buffer. Animals were resuspended in ice-cold RIPA buffer (BioBasic, RB4478) containing a protease inhibitor cocktail (Roche, 11,836,153,001) (1 mL of buffer per 0.1 g of *C. elegans* pellet) and then sonicated at 70% power for 2 runs of 10 s each (Qsonica, Q125). Sonicated samples were incubated at 4 °C for 2 h in a shaker and then centrifuged at 17,000 g for 30 min. ELISAs were performed to measure α-synuclein protein levels in the protein lysate (supernatant). Total protein was quantified using a commercial BCA assay (ThermoFisher, 23,227) according to the manufacturer’s instructions. ELISAs were conducted using a commercial human α-synuclein ELISA kit (BioLegend, 844,101) according to the manufacturer’s instructions.

### Dopaminergic neuron loss in *C. elegans*

Control (*tzIs3;egIs1*) and α-synuclein ([A30P α-synuclein];*tzIs3;egIs1*) gravid adult animals were synchronized, and eggs were hatched overnight (day 0) on NGM plates without food. Synchronized L1 larvae were transferred to NGM plates with food and grown at room temperature. L4 larval animals were then picked and transferred to fresh NGM plates with food for better synchronization. The animals were transferred to new plates every 48 to 72 h. On day 3 to day 9, anterior deirid (ADE) neurons were scored from 26 to 58 animals in a blinded fashion by GFP fluorescence using a widefield microscope (Zeiss AxioObserver). Any absent or unidentifiable ADE neuron was scored as degenerated.

### CRE-GFP reporter of dopaminergic dysfunction in *C. elegans*

Animals were synchronized by isolating eggs using alkaline hypochlorite treatment and hatching overnight. Larvae were plated onto NGM agar plates with OP50 and grown for 3 to 4 days prior to CRE-GFP analysis. Animals were washed with M9 buffer until clear of bacteria and then resuspended in a small volume of M9 buffer. Animals were mounted on 10% agarose pads and immobilized on the pads with 0.2 to 0.5 μl of 0.1 μm diameter polystyrene microspheres (Polysciences, 00876–15). GFP fluorescence of 4 cholinergic head neurons named SIA (sublateral interneuron) was scored in a blinded fashion using a widefield fluorescence microscope (Zeiss AxioObserver). We recorded the number of animals with at least one GFP positive SIA neuron, as well as the number of GFP positive SIA neurons per animal.

### Gene ontology (GO) analysis of *C. elegans* coiler strains

Yemini et al. [16] published a database of behavioural phenotypes for 305 *C. elegans* strains, including the coiler phenotype (reported as coiling frequency and coil time). Ninety-seven genes were associated with *C. elegans* strains having a statistically significant increase in either coiler frequency or coil time compared to N2 (binned q value of < 0.05). These genes were analysed with PANTHER gene ontology “GO biological process complete” (pantherdb.org/geneListAnalysis.do). The *C. elegans* genome was used as the reference list. For all GO terms > 100-fold enrichment, we ranked the biological processes by the -log10 of the false discovery rate (FDR).

### Immunoprecipitation and immunoblotting

Synchronized animals (4 or 5 days old) were lysed in 0.5X Tris-HCl buffer with 0.5% Triton X-100 at 100 °C for 5 min and then passed through a 23-gauge needle 10 times. The lysate was centrifuged at 10,000 g for 10 min in a 4 °C refrigerated centrifuge, and the protein lysate (supernatant) was transferred to a new tube. Protein concentration was determined with a Bio-Rad Lowry protein assay (Bio-RAD, 5000116) according to the manufacturer’s instructions. For immunoprecipitation, protein lysates were incubated on a rotator at 4 °C overnight with mouse anti-human α-synuclein (Clone 42, BD Laboratories, 610,787; dilution 1:100). Immune complexes were isolated by the addition of washed protein G agarose beads followed by incubation for 4 h at 4 °C. Beads were washed and samples were analysed by immunoblotting and mass spectrometry (see below). For immunoblotting, protein lysates (9.5 μg) or beads from immunoprecipitations were mixed with 6X Laemmli sample buffer, and proteins were then separated on a 10% acrylamide gel by SDS-PAGE. Proteins were transferred to a PVDF membrane using a wet transfer system. The membranes were probed with anti-α-synuclein mouse monoclonal antibodies (Clone 42, BD Laboratories, 610,787; dilution 1:500). Biotinylated goat anti-mouse antibodies (Jackson Immuno Research, 115,065,146; dilution 1:20,000) were used as secondary antibodies with streptavidin-conjugated with horseradish peroxidase (Jackson Immuno Research, 016030084; dilution 1:10,000). Signals were detected with ECL (ThermoFisher, 32,132) and developed on HyBlot CL autoradiography film (DV-E3018).

### Mass spectrometry and GO analysis of α-synuclein-interacting *C. elegans* proteins

Liquid chromatography-tandem mass spectrometry (LC-MS/MS) was performed by SPARC BioCentre Molecular Analysis, The Hospital for Sick Children, Toronto, Canada using samples of proteins co-immunoprecipitated with α-synuclein (described above). One hundred and thirty-one proteins were identified in immunoprecipitants from α-synuclein *C. elegans* lysates but absent from GFP *C. elegans* lysates. The genes that encode these proteins were analysed with PANTHER gene ontology “GO biological process complete” (pantherdb.org/geneListAnalysis.do). The *C. elegans* genome was used as the reference list. Biological processes with FDR < 0.05 were ranked according to fold enrichment.

### In silico ranking of drugs predicted to reduce α-synuclein oligomers

IBM Watson for Drug Discovery Predictive Analytics identified ~ 26 million records in Medline that cited either the known or candidate entities, as we previously described [17]. Every single Medline abstract was then converted into a multidimensional vector of the words and phrases contained in the document, relative to the 20,000 most common words and phrases within the English lexicon, using a term frequency–inverse document frequency statistic. A centroid for each known and candidate entity was then generated by averaging the multidimensional vectors of all documents associated with each entity and used to produce a distance matrix comprising a similarity index for every pair of entities. Finally, a graph diffusion algorithm was applied to rank each candidate entity by similarity to the entire known set rank, thus producing a ranked candidate list ordered by predicted semantic similarity to the known set. The model performance was validated using a leave-one-out (LOO) cross-validation in which the ranking process (as described above) was run 15 times, each time with one entity removed from the known set and added to the candidate set. Receiver-Operating Characteristic (ROC) and Precision-Recall curves were generated.

### Luciferase protein-fragment complementation and cell viability assays in cell lines

H4 neuroglioma cells (ATCC, HTB-148) were maintained in Dulbecco’s Modified Eagle Medium plus high glucose, L-glutamine, and sodium pyruvate (ThermoFisher, 11995–065) with 10% heat-inactivated fetal bovine serum (ThermoFisher, 16140071) and 1% antibiotic-antimycotic cocktail (ThermoFisher, 15240062) at 37 °C and 5% CO_2_. DNA expression constructs encoding for full-length human α-synuclein fused to the N-terminal fragment of *Gaussia princeps* luciferase (syn-luc1), full-length human α-synuclein fused to the C-terminal fragment of *Gaussia princeps* luciferase (syn-luc2), and full-length *Gaussia princeps* luciferase were kindly provided by Dr. Pamela McLean, Mayo Clinic Jacksonville [18]. DNA expression constructs encoding for human amyloid-beta peptide Aβ1–42 fused to the N-terminal fragment of *Gaussia princeps* luciferase (Aβ-luci) and human amyloid-beta peptide Aβ1–42 fused to the C-terminal fragment of *Gaussia princeps* luciferase (Aβ-ferase) were kindly provided by Dr. Tadafumi Hashimoto, University of Tokyo [19]. A DNA expression construct encoding for full-length human tau protein (2 N, 4R) fused to the C-terminal fragment of *Gaussia princeps* luciferase (tau-ferase) was kindly provided by Dr. Susanne Wegmann, DZNE Berlin [20]. To generate a construct encoding for full-length human tau protein (2 N, 4R) fused to the N-terminal fragment of *Gaussia princeps* luciferase (tau-luci), the luciferase fragment from luci-Aβ was PCR amplified using Q5 polymerase (New England Biolabs) and the primers: BspeILUCIf 5′ GTGGGTCCTCCGGAAAGCCCACCGAGAACAACGAAGACTTCAAC 3′ and KpnILUCIr 5’ATCGGATCCGGTACCGATTTAAACGGGCCCTCTAGATTAGCCTATGCCGCCCTG3’. The fragment was cloned into the Bspe I/ Kpn I sites of tau-L2 to produce tau-luci (pAK 348–1) which was sequenced to confirm accuracy and freedom from PCR induced errors. Cells were transiently co-transfected with syn-luc1 and syn-luc2, Aβ-luci and Aβ-ferase, tau-luci and tau-ferase, or transfected with luciferase alone using Superfect transfection reagent (Qiagen, 301305) according to the manufacturer’s instructions. Twenty-four hours after transfection, cells were plated on a 96-well microplate at 60,000 cells per well. Twenty-four hours after plating, cells were treated with drugs or vehicle control. Following 24 h of treatment, cells were washed 1 time with PBS and bioluminescence was measured using an automated CLARIOstar plate reader (Mandel, 430–0505). Cell permeable, native coelenterazine was used as the *Gaussia* luciferase substrate. Lyophilized coelenterazine (Nanolight, 303–500) was reconstituted in NanoFuel Solvent (Nanolight, 399–1), then diluted in PBS to 16.6 μg/mL, and dispensed per well to a final concentration of 20 μM). The bioluminescent signal generated by the luciferase enzyme was assessed at 470 nm over 5 s. Raw luminescence units were normalized to the DMSO vehicle control of each 96-well microplate. To assess cell viability, H4 neuroglioma cells were prepared and treated as described above and, 1 h prior to viability analysis, cells were treated with PrestoBlue viability reagent (ThermoFisher, A13261) according to the manufacturer’s instructions. Fluorescence was automatically measured using a CLARIOstar plate reader (Mandel, 430–0505) and then normalized to the fluorescence measure of the DMSO vehicle control.

### Venus YFP protein-fragment complementation assay in primary cortical neurons

Pregnant Sprague Dawley rats (E17) were purchased from Charles River. Embryos were surgically removed, and neurons were harvested in a sterile environment. Neurons were plated on poly-D-lysine coated glass coverslips at a density of 5 × 10^5^ cells per well in Neurobasal-A medium, (Gibco, 10,888,022) supplemented with B27 (2%) (Gibco, 17,504,044), antibiotic-antimycotic (1%) (Gibco, 15,240,062), and Glutamax (1%) (Gibco, 35,050,061). Fifty percent media changes were performed every 3 days. DNA expression constructs encoding for full-length human α-synuclein fused to the N-terminal fragment of Venus YFP (V1S), full-length human α-synuclein fused to the C-terminal fragment of Venus YFP (SV2), and full-length Venus YFP were kindly provided by Dr. Pamela McLean, Mayo Clinic Jacksonville [21]. The following AAV serotype 1/2 vectors were custom ordered from GeneDetect (Auckland, New Zealand): AAV1/2-CBA-V1S-WPRE-BGH-polyA (AAV-V1S), AAV1/2-CBA-SV2-WPRE-BGH-polyA (AAV-SV2), and AAV1/2-CBA-Venus YFP-WPRE-BGH-polyA (AAV-Venus). Two days post isolation, neurons were co-transduced with AAV-V1S and AAV-SV2 or transduced with AAV-Venus alone at a MOI of 3000. AAV-containing media was removed after 72 h and cells were then treated with drugs or DMSO control for 72 h at the following concentrations: acetaminophen (100 μM), caffeine (200 μM), losartan (100 μM), rapamycin (50 nM), rifabutin (20 μM). Concentrations were selected to avoid neuronal toxicity according to previous reports [22–26]. Drug-containing media was removed, and cells were washed 1 time with ice-cold PBS before being fixed with 4% PFA (Sigma) at room temperature for 10 min. Levels of α-synuclein oligomers were measured by YFP fluorescence. To measure total α-synuclein levels, neurons were immunostained with anti-α-synuclein mouse monoclonal antibodies (Syn211, ThermoFisher, 32–8100; dilution 1:500) and goat anti-mouse secondary antibodies conjugated to Alexa Fluor® Plus 555 (ThermoFisher, A32727; dilution 1:500). Images of fixed cells were acquired using a confocal microscope equipped with 405, 488, 555, and 639 nm laser lines (Zeiss LSM880). All images for each biological replicate were taken within the linear range at constant gain and pinhole settings at a resolution of 1024 × 1024 pixels using a 63x/NA1.4 oil objective with the zoom set to 1.2. The imaging medium used was Zeiss Immersol 518F. Fluorescence intensity was analysed automatically using Imaris imaging software (Oxford Instruments) by measuring the mean fluorescence intensity of each individual fluorescent neuron throughout a 3D field of view generated by z-stack image acquisition with the “surfaces” module. This module detects fluorescence above background threshold in each field of view, identifies a cell body and neuronal projections of each cell, and calculates the mean fluorescence intensity of each fluorescent cell within the field of view. The mean fluorescence intensity of 10 fields of view was recorded for each biological replicate and normalized to the mean intensity of the DMSO treated condition. The value generated for each neuron was normalized to the mean of fluorescence intensity of neurons treated with vehicle alone.

For crosslinking experiments, primary cortical neurons were cultured and transduced as above. Eight days post isolation, media was removed, and cells were washed twice in PBS before being scraped and centrifuged at 1200 g for 5 min. Cells were re-suspended in PBS containing 50 μM disuccinimidyl glutarate (DSG; ThermoFisher, 20,593) and incubated at 37 °C for 30 min according to the manufacturer’s instructions. The reaction was quenched by the addition of 50 mM Tris for 15 min. Cells were pelleted again and re-suspended in RIPA buffer and prepared for SDS-PAGE.

### Stereotactic surgery and rifabutin treatment of rats

Adult female Sprague-Dawley rats (250–300 g) were purchased from Envigo. The animals were pair-housed in cages with wood bedding and access to food and water ad libitum. The animal colony was maintained in a regular 12-h light/dark cycle. All procedures were approved by the University Health Network Animal Care Committee in accordance with guidelines and regulations set by the Canadian Council on Animal Care.

AAV serotype 1/2 was used to express A53T α-synuclein (AAV-A53T) under the control of the CAG promoter, a hybrid of the chicken beta actin (CBA) promoter fused with the cytomegalovirus (CMV) immediate early enhancer sequence (GeneDetect, Auckland, New Zealand), as previously described [27]. An AAV1/2 vector lacking the A53T α-synuclein open reading frame was used as an empty vector control (AAV-EV). Animals were secured in a stereotactic frame under isoflurane/oxygen anaesthesia (2.5% isoflurane and 1.5 L/min O_2_) and anafen (5 mg/kg) analgesia. The surgical site was shaved and sterilized with iodine/betadine/isopropanol prior to making a 2-cm incision along the midline. The skull was exposed and a unilateral injection targeting the SN was performed at coordinates AP − 5.2 mm, ML − 2 mm, and DV − 7.5 mm with respect to bregma. For each animal, a total volume of 1.5 μl of AAV-A53T or AAV-EV (5.1 × 10^12^ genomic particles/ml) plus 0.5 µl of sterile PBS was injected at a rate of 0.5 μl/min using a microinjection pump and 10 μl Hamilton syringe with a 26-gauge needle. At the end of virus injection, the needle remained in place for 5 min before gradual removal.

Rats were randomly assigned to receive 25 mg/kg rifabutin (prepared as 1.5 mg/ml in 5% DMSO in saline) or vehicle control (5% DMSO in saline) daily. Treatments were started 2 days following stereotactic surgery. Rats were weighed and treated each day between 7:00–8:00 am with rifabutin or vehicle by intraperitoneal injection using a 25-gauge needle for 6 weeks.

### Collection of rat plasma, cerebrospinal fluid, and brain tissue

Animals were euthanised after 6 weeks of rifabutin or vehicle treatment by cardiac puncture under isoflurane/oxygen anaesthesia, followed by transcardial perfusion with approximately 100 to 200 ml of ice-cold heparinised saline. Blood obtained via cardiac puncture was centrifuged at 4 °C in microtainer tubes (BD, 365974) for 2 min at 10,000 g, and the upper layer plasma was collected, frozen on dry ice, and stored at − 80 °C until use. Cerebrospinal fluid (CSF) was collected using a latex dropper bulb attached to a custom-made glass micropipette (Drummond Scientific, Broomall, PA) inserted into the cisterna magna. Approximately 50 to 100 μl of CSF were transferred into autoclaved vials and centrifuged at 3000 rpm over 3 to 5 s; samples with a detectable red blood cell precipitate were excluded due to blood contamination. CSF samples were frozen on dry ice and stored at − 80 °C until use. Brains were then removed, and tissue anterior to the optic chiasm was snap frozen in dry ice-cooled isopentane. A single 1 mm thick section of the ventral striatum was immediately cut, using a matrix, and frozen on dry ice. These sections were sent to Vanderbilt University Neurochemistry Core (Nashville, TN, USA) for measurements of biogenic amines by high-performance liquid chromatography (HPLC). Approximately 100 mg of frozen brain tissue plus frozen plasma samples from a subset of animals were sent to InterVivo Solutions (Toronto, ON, Canada) for measurements of rifabutin concentrations by LC-MS/MS. Tissue posterior to the optic chiasm, including the posterior striatum and SN, was immersion-fixed in 4% paraformaldehyde in 0.1 M PBS for 2 days at room temperature and cryoprotected at 4 °C in 15% sucrose and then 30% sucrose in 0.1 M PBS solution until the brains sank. For immunostaining, 40 μm thick coronal cryosections were prepared using a sliding microtome (Leica Microsystems Inc.), and 6 series of sections were stored in cryoprotectant (30% glycerol, 30% ethylene glycol, 40% PBS) at − 20 °C until use.

### Immunostaining of brain cryosections

Immunostaining for stereology was performed by washing free-floating sections with PBS-T (PBS with 0.1% Tween-20) three times for 10 min each at room temperature. Sections were then immersed in 3% H_2_O_2_ for 3 min to quench endogenous peroxidases. Sections were rinsed in PBS-T three times for 5 min each before incubation in blocking solution (2% BSA, 10% normal goat serum in PBS-T) for 1 h at room temperature. After blocking, sections were incubated with rabbit anti-tyrosine hydroxylase (TH) antibodies (ThermoFisher Scientific, AB152; dilution 1:2000) in blocking solution overnight at room temperature. Sections were washed in TBS-T (TBS with 0.1% Tween-20) before incubation with alkaline phosphatase-conjugated goat anti-rabbit (H + L) secondary antibodies (Jackson ImmunoResearch, 111–055-144; dilution 1:500) in 2% NGS TBS-T for 2 h at room temperature. Sections were then washed three times for 5 min each in TBS-T before incubation in Vector Blue substrate, prepared by adding 2 drops of reagents 1, 2, and 3 to 5 ml of 100 mM Tris-HCl pH 8.2 (Alkaline Phosphatase Substrate Kit III, Vector Labs, SK-5003). The reaction was stopped by incubation of sections in 100 mM Tris-HCl pH 8.2 before the sections were washed five times for 3 min each in PBS. Sections were mounted onto slides and allowed to air-dry overnight. Slides were dehydrated by incubating for 3 min in ddH_2_O, then for 1 min each in 70, 95, and 100% EtOH, and finally two times for 3 min each in Histoclear (Harleco, 65,351). Vectamount (Vector Labs, H-5000) was applied prior to coverslip application.

Immunofluorescent staining was performed by washing free-floating sections with PBS-T (0.2% Tween-20 or 0.1% Triton X-100) three times for 5 or 10 min each at room temperature. To detect total α-synuclein, sections were then incubated in blocking solution (2% BSA, 10% normal goat serum in PBS-T) for 1 h at room temperature. After blocking, sections were incubated with rabbit anti-TH antibodies (ThermoFisher Scientific, AB152; dilution 1:1000) and anti-α-synuclein mouse monoclonal antibodies specific for human α-synuclein (Syn211, ThermoFisher, 32–8100; dilution 1:500) in antibody solution (2% normal goat serum in PBS-T) overnight at room temperature. Sections were washed in PBS-T and incubated with secondary fluorescent antibodies in antibody solution for 1 h in the dark at room temperature. Secondary antibodies were Alexa Fluor goat anti-rabbit 594 (Invitrogen, A11037; dilution 1:500) and Alexa Fluor goat anti-mouse 488 (Invitrogen, A11029; dilution 1:500). To detect α-synuclein oligomers, sections were treated with 1 M glycine for 30 min and then incubated in blocking solution (1% BSA, 10% normal goat serum in PBS-T) for 1 h at room temperature. After blocking, sections were incubated with chicken anti-TH antibodies (Abcam, ab76442; dilution 1:1000) and Syn-O2 antibodies (dilution 1:5000) in blocking solution overnight at room temperature. Syn-O2 is a mouse monoclonal antibody which specifically recognizes early soluble oligomers and late fibrils of α-synuclein, and Syn-O2 has a high binding affinity for oligomeric α-synuclein [28]. Sections were washed in PBS and incubated with biotin-conjugated goat anti-mouse secondary antibodies (Jackson ImmunoResearch, 115–065-146; dilution 1:500) in PBS for 1 h at room temperature. Sections were washed in PBS and incubated with Alexa Fluor goat anti-chicken 594 (Invitrogen, A11042; dilution 1:500) and Alexa Fluor 488 streptavidin (Invitrogen, S32354; dilution 1:500) in PBS for 1 h in the dark at room temperature. Sections were washed in PBS, mounted onto glass slides, and allowed to dry. Fluorescence mounting medium (DAKO) was applied, followed by coverslip application. Appropriate targeting of AAV-A53T injection in the SN was based on the findings from immunostaining human α-synuclein; animals were considered mistargeted and thus excluded from analyses if their SN cells were not transduced with AAV-A53T.

### Image analysis of brain cryosections

Imaging analyses were performed by a researcher blinded to the drug treatments. Confocal images of immunofluorescent staining were acquired with a Zeiss LSM700 confocal microscope equipped with 405, 488, 555, and 639 nm laser lines. All images were taken within the linear range at constant gain and the pinhole settings at optimal resolution settings determined by the software. The whole midbrain regions were imaged using a 10X objective. Four serial coronal midbrain sections were imaged per animal, separated by 240 μm intervals. Confocal images of immunofluorescent stained midbrain sections were processed using HALO software (Indica Labs). Injected SN was selected as a region of interest (ROI), and dopaminergic neurons were identified by automated detection of TH-labelled cells within this ROI. Dopaminergic neuron densities, which correlate with neuronal counts obtained by conventional stereology as previously described [29], were estimated using HALO software. Levels of total human α-synuclein (detected by Syn211 staining) or α-synuclein oligomers (detected by Syn-O2 staining) were assessed in this ROI by measuring fluorescence intensity.

### Stereology of brain cryosections

The optical fractionator method was used for the unbiased stereological estimation of dopaminergic (i.e., TH-positive) cell counts in the injected and non-injected SN (Stereo Investigator software version 9, MBF Biosciences). The investigator was blinded to the experimental groups. Every sixth section throughout the SN was quantified (7 sections total). The injected or non-injected SN was selected as a ROI bounded at a 10x objective, and counting was performed using a 40x oil-immersion objective. A guard zone thickness of 2 μm was set at the top and bottom of each section. The sampling interval in the X-Y coordinate axis was set as follows: 175 μm × 175 μm counting frame size; 300 μm × 200 μm grid size; 20 μm dissector height. Coefficient of error was calculated according to Gundersen and Jensen [30], and values < 0.10 were accepted.

### Real-time quaking induced conversion (RT-QuIC) assay

A modified version of the α-synuclein RT-QuIC assay described by Barger et al. [31] was used. Dilution of rat CSF (1:10) was performed with 1x N2 supplement (Thermo Fisher) diluted with PBS. Black 96-well, clear bottom plates were pre-loaded with six 1 mm in diameter silica beads per well (OPS Diagnostics). Lyophilized recombinant human α-synuclein (Sigma) was reconstituted to 1 mg/ml with filtered ddH_2_O and centrifuged for 10 min at 4 °C through 100 kDa Amicon filters (Millipore). Diluted rat CSF (15 μl) was added to individual wells containing 85 μl of RT-QuIC reaction mixture composed of 40 mM NaPO_4_ pH 8.0 (Boston BioProducts), 170 mM NaCl, 0.1 mg/ml recombinant α-synuclein, 20 μM ThT (Sigma), and 0.0005% SDS. The plates were sealed with clear plastic film (Thermo Fisher) and incubated at 42 °C in Clariostar plate reader (BMG Labtech) with cycles of 1 min shaking (400 rpm, double orbital) and 1 min rest throughout the indicated incubation time. ThT fluorescence (450 nm excitation and 480 nm emission) was recorded every 45 min for 60 h. Replicate reactions were run in four separate wells for each sample. Positive RT-QuIC reactivity of individual wells was defined as enhanced ThT fluorescence above a predefined threshold at 60 h. This threshold was calculated as the mean background fluorescence of the diluent plus 5 standard deviations, equal to approximately 3200 relative fluorescence units (rfu) in our experiments. A sample was considered positive when at least one of the four replicates displayed positive ThT reactivity above the threshold. For each positive sample, the mean fluorescence intensity from the positive replicates was calculated and plotted against time. For each negative sample, the mean fluorescence intensity from the negative replicates was calculated and plotted against time.

### Statistical analysis

All statistical analyses were conducted using GraphPad Prism 6 or 8. Mean ± SEM with individual replicates are presented when appropriate. The specific statistical tests performed are indicated.

## Results

### Mild coiling is a motor impairment of *C. elegans* expressing mutant α-synuclein

**Fig. 1:**
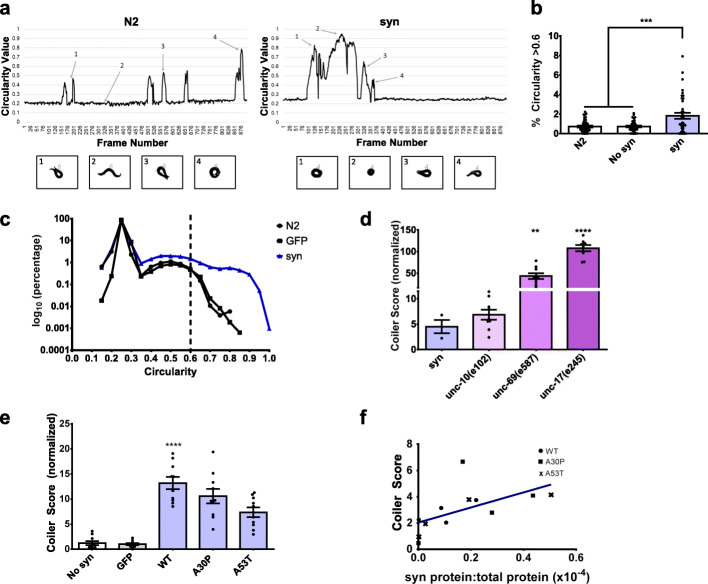
Coiling is a motor impairment of *C. elegans* that correlates with α-synuclein protein levels. (a) *Top:* Circularity values per frame from recordings of individual non-transgenic *C. elegans* (N2 wild-type strain) or transgenic *C. elegans* expressing A30P α-synuclein in dopaminergic neurons (syn). *Bottom:* Processed images (numbered 1 to 4) of the animal corresponding to different circularity values. Normal sinusoidal body shape corresponded to circularity values of ~ 0.2 (left, image 2). Body shape during an omega turn corresponded to circularity values of ~ 0.5 (left, images 1 and 3; right, image 4). Coiling animals had circularity values ranging from ~ 0.6 to almost 1 (left, image 4; right, images 1 and 2). (b) Percentage of frames with a circularity value > 0.6 for individual N2 (*N* = 40 animals), syn (*N =* 40 animals), or animals that do not express α-synuclein but have the same genetic background (*tzIs3*) as syn animals (No syn) (*N* = 41 animals) (one-way ANOVA with Tukey’s post-hoc test, ***p < 0.001). Each data point represents an individual animal. (c) Recordings were performed of N2, syn, or control animals expressing GFP instead of α-synuclein in dopaminergic neurons (*N* > 10 populations of *n* = 10 animals per strain), and circularity values were calculated for each animal in each frame. Range of circularity values was divided into bins of 0.05 (20 bins from 0 to 1.0). Number of circularity values within each bin were counted and percentage relative to all counts was calculated (presented as log_10_ of percentage). Circularity value of 0.6 (dotted line) was selected as the threshold to isolate the coiler phenotype. (d) Coiler scores of syn animals (*N* = 3 populations of *n =* 10 animals) and known coiler mutant strains: *unc-10(e102)* (*N* = 9 populations of *n =* 10 animals), *unc-69(e587)* (*N =* 9 populations of *n =* 10 animals), and *unc-17(e245)* (*N =* 9 populations of *n =* 10 animals), normalized to control GFP animals (one-way ANOVA with Tukey’s post-hoc test, **p < 0.01, ****p < 0.0001 relative to syn animals). Each data point represents an individual population. (e) Coiler scores of animals expressing wild-type (WT), mutant A30P, or mutant A53T α-synuclein in dopaminergic neurons. Control *C. elegans* express GFP only in dopaminergic neurons (GFP) or do not express either α-synuclein or GFP (No syn). Coiler scores are normalized to control GFP animals (*N =* 10 populations of *n* = 10 animals for each strain; each data point represents an individual population) (one-way ANOVA with Tukey’s post-hoc test, ****p < 0.0001 relative to GFP). (f) Correlation of coiler score and α-synuclein protein levels for 12 different *C. elegans* strains expressing α-synuclein in dopaminergic neurons (Pearson correlation, r = 0.58, *p* < 0.05). Each point of the scatter plot represents one animal strain. The syn protein:total protein ratio for the 3 lowest expressing strains ranged from 0.03 to 0.2 × 10^− 6^

Motor behaviour is a principal output of the *C. elegans* nervous system and consequently neural perturbations are frequently associated with changes in locomotion [16]. Yet, expression of α-synuclein in dopaminergic neurons of *C. elegans* has not been previously reported to cause visible defects in spontaneous crawling. We observed a subtle uncoordinated (*unc*) “coiler” phenotype among transgenic *C. elegans* expressing an untagged mutant form of human α-synuclein, with alanine at amino acid position 30 mutated to proline (A30P), under control of the dopamine neuron specific promoter, *dat-1p*. The A30P mutation is a known cause of inherited monogenic PD [2]. Coiling is an abnormal motor behaviour in which the animal spends increased time with its body in a round or circular position. To measure this motor impairment, we used a custom-built tracking microscope that captures and follows the motions of single *C. elegans* with high spatial and temporal resolution to record their locomotion [32]. We quantified coiling by calculating a numerical value for circularity, which corresponds with *C. elegans* adopting a circular shape. The circularity value is based on the formula 4π(A/P^2^), where A is area and P is perimeter of the worm’s body [33]. A value of 1 indicates a perfect circle and a value approaching 0 indicates an increasingly elongated shape. Recordings of individual transgenic *C. elegans* expressing A30P α-synuclein or non-transgenic *C. elegans* (N2 wild-type strain) revealed that the normal sinusoidal shape of the animals corresponded to circularity values of ~ 0.2. The body shape during an omega turn, which is a normal motor behaviour used by the animal to change directions, corresponded to circularity values of ~ 0.5, whereas coiling animals had circularity values ranging from ~ 0.6 to almost 1 (Fig. 1a). We found that A30P α-synuclein *C. elegans* had circularity values in the coiling range for a greater proportion of video frames than non-transgenic N2 animals or *C. elegans* with the same genetic background (*tzIs3*) but expressing no α-synuclein (Fig. 1b). Further, N2 and *tzIs3* animals showed the same degree of circularity, suggesting that *tzIs3* did not affect coiling.

We next devised a method to quantify coiling for a population of animals. Specifically, using high-resolution, multi-worm tracking with custom image processing, we developed methods for semi-automated and blinded scoring of circularity for a population of simultaneously recorded animals (*n* = 10 per recording). A coiler score was determined for the population tested by first calculating the circularity value for each animal in each frame of video and then calculating the proportion of circularity values > 0.6. A circularity value threshold of 0.6 was chosen to isolate the coiler phenotype because we found that, for N2 animals or control animals expressing green fluorescent protein (GFP) instead of α-synuclein in dopaminergic neurons, the likelihood of circularity values > 0.6 was very low compared to untagged A30P α-synuclein animals (Fig. 1c). The coiler phenotype is an abnormal motor behaviour previously reported for several *unc C. elegans* strains with mutations affecting neuronal function. To test the validity of our methods for scoring the coiler phenotype, we examined three *C. elegans* strains with differing degrees of coiling due to presynaptic dysfunction: 1) *unc-10(e102)*, a mild coiler with a mutation in an ortholog of human RIMS1 (regulating synaptic membrane exocytosis 1) which is involved in presynaptic vesicular priming [34]; 2) *unc-69(e587)*, a moderate coiler with a mutation in an ortholog of human SCOCO (short coiled-coil protein) which functions in axonal outgrowth and presynaptic organization [35]; and, 3) *unc-17(e245)*, a severe coiler with a mutation in an ortholog of human SLC18A3 (solute carrier family 18 member A3) which exhibits acetylcholine transmembrane transporter activity [36]. We found the mean + SEM coiler scores for *unc-10(e102)*, *unc-69(e587)*, and *unc-17(e245)*, normalized to control GFP animals, to be 6.7 + 1.0, 43.2 + 6.6, and 107.8 + 7.0, respectively (Fig. 1d). The normalized mean + SEM coiler score for animals expressing A30P α-synuclein was 4.5 + 1.3, similar to that of *unc-10(e102)*. These results demonstrate that our methods can quantify the coiler phenotype of a *C. elegans* population and appropriately differentiate between different degrees of coiling. Therefore, our findings suggest that mild coiling is a motor phenotype in *C. elegans* expressing untagged A30P α-synuclein exclusively in dopaminergic neurons which can be measured using our methods for semi-automated and blinded scoring of a population of animals.

### Coiling correlates with mutant or wild-type α-synuclein protein levels

To determine whether the coiler phenotype we discovered for *C. elegans* expressing mutant A30P α-synuclein was a motor abnormality of other transgenic α-synuclein *C. elegans*, we also examined the locomotion of *C. elegans* expressing untagged mutant A53T or wild-type α-synuclein only in dopaminergic neurons. Transgenic A30P, A53T, and wild-type α-synuclein *C. elegans* demonstrated comparable coiler scores which were each higher than the coiler score for control *C. elegans* expressing GFP instead of α-synuclein in dopaminergic neurons or animals without expression of ectopic protein in those neurons (Fig. 1e).

A decline in locomotion occurs as *C. elegans* age and hence motor impairment may correlate with the animal’s lifespan. For example, *C. elegans* with mutations that prolong lifespan have slower motor decline, whereas the decline is accelerated in animals with mutations that reduce longevity [37]. Since ectopic expression of α-synuclein in *C. elegans* has been reported to have variable effects on lifespan [38–40], we measured lifespans of the transgenic A30P, A53T, and wild-type α-synuclein *C. elegans*. Their mean (+ SD) lifespans were comparable at 16.3 + 0.5, 16.1 + 1.2, and 17.9 + 1.2 days, respectively, and were not statistically significantly shorter than that of control GFP animals (16.7 + 0.9 days). Thus, a shorter lifespan could not account for the motor dysfunction observed in these transgenic α-synuclein *C. elegans*.

Because the copy number of transgenes can vary between each *C. elegans* line, we tested whether the differences in coiling between the transgenic α-synuclein animals were related to α-synuclein protein expression levels. We measured the coiling behaviour and α-synuclein protein levels of 12 independent transgenic *C. elegans* lines: 9 expressing α-synuclein in dopaminergic neurons, and 3 co-expressing α-synuclein and GFP in dopaminergic neurons. We observed a moderate positive correlation between coiling severity and α-synuclein protein expression (Fig. 1f). Together, these findings indicate that coiling is not simply an artefact of ectopic protein expression in dopaminergic neurons since this motor phenotype occurred with α-synuclein but not GFP. Analogous to PD in humans, a more severe phenotype is associated with higher expression of α-synuclein [41]. We found that expression in dopaminergic neurons of either mutant α-synuclein (associated with familial PD) or wild-type α-synuclein (associated with sporadic PD) caused coiling. Therefore, for the remainder of the present study, we used the transgenic A30P α-synuclein *C. elegans*.

### Coiling results from dopaminergic neuron dysfunction due to α-synuclein and precedes neuronal loss

**Fig. 2:**
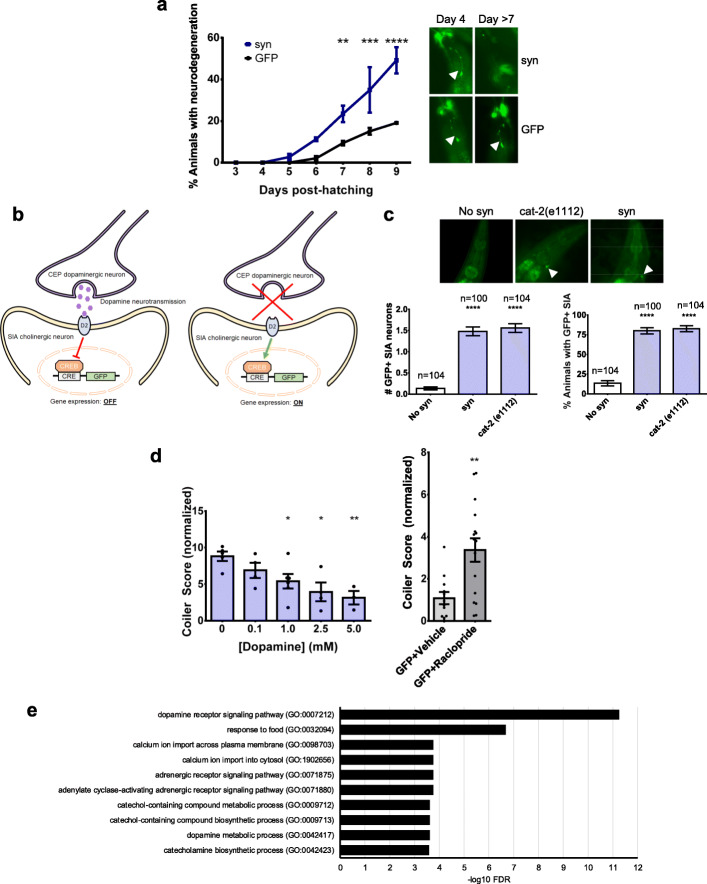
Coiling results from early dopaminergic neuron dysfunction due to α-synuclein preceding neuronal loss. (a) *Left:* Loss of dopaminergic ADE neurons over time for *C. elegans* expressing A30P α-synuclein plus GFP in dopaminergic neurons (*N =* 3 populations of *n* = 50 animals) and for *C. elegans* expressing GFP alone (*N* = 4 populations of *n =* 50 animals) (two-way ANOVA with Bonferroni’s post-hoc test, **p < 0.01, ***p < 0.001, ****p < 0.0001 comparing syn plus GFP and GFP alone animals on the same experimental day). Each data point represents an individual population. *Right:* Representative fluorescent microscopy images of surviving ADE dopaminergic neurons (arrowheads). (b) CRE-GFP reporter in SIA cholinergic neurons is used to assess for intact (GFP expression “OFF”) or disrupted (GFP expression “ON”) presynaptic signalling from the upstream CEP dopaminergic neuron. (c) *Top:* Representative fluorescent microscopy images of GFP positive SIA cholinergic neurons (arrowheads). *Bottom:* Quantification of number of GFP positive SIA neurons (left graph) and percentage of animals with GFP positive SIA neurons (right graph) for control animals not expressing α-synuclein (No syn) (*N* = 104 animals), syn animals (*N* = 100 animals), and a tyrosine hydroxylase mutant strain *cat-2 (e1112)* (*N =* 104 animals) (one-way ANOVA with Tukey’s post-hoc test, ****p < 0.0001). (d) *Left:* Coiler scores of syn animals treated with increasing concentrations of exogenous dopamine, normalized to vehicle treated control GFP animals (*N* = 3 populations of *n* = 10 animals per treatment condition) (one-way ANOVA with Dunnett’s post-hoc test, *p < 0.05, **p < 0.01 relative to [Dopamine] 0 mM). *Right:* Coiler scores of control GFP animals treated with 1 mM raclopride (*N* = 16 populations of *n =* 10 animals) or vehicle (*N* = 12 populations of *n =* 10 animals), normalized to vehicle treated control GFP animals (two-tailed t-test, **p < 0.01). Each data point represents an individual population. (e) Gene ontology enrichment analysis of biological processes implicated in coiling using 97 genes from *C. elegans* strains previously reported to demonstrate coiling behaviour. Biological processes over-represented by a factor of > 100 were ranked according to the –log_10_ false discovery rate

Prior to the development of transgenic animals to model PD according to monogenic causes of the disease, most PD animal models were based on treatment with neurotoxic chemicals [42]. A commonly used model involved treatment with 1-methyl-4-phenyl-1,2,3,6-tetrahydropyridine (MPTP), which is known to induce severe PD symptoms in humans [43]. When administered to mammals, MPTP crosses the blood-brain barrier and is converted in the brain to its toxic metabolite 1-methyl-4-phenylpyridinium (MPP+), which causes selective degeneration of dopaminergic neurons in the SN by inhibiting the respiratory chain enzyme complex I in mitochondria. Treatment of *C. elegans* with MPP+ has also been reported to cause selective loss of dopaminergic neurons plus motor defects that include coiling [44]. Since our intent was to develop a model that recapitulates early stages of neurodegeneration, we investigated whether coiling of transgenic A30P α-synuclein *C. elegans* occurred prior to dopaminergic neuron loss, which is typical of late stage disease. A *C. elegans* hermaphrodite has 8 dopaminergic neurons, each with a stereotyped location: 2 pairs of cephalic (CEP) neurons and 1 pair of anterior deirid (ADE) neurons in the head region, and 1 pair of posterior deirid (PDE) neurons in the posterior body region. Because *C. elegans* are transparent, fluorescent markers are easily visualized in vivo, and loss of fluorescence is an indirect but frequently used indicator of neurodegeneration [9, 10]. We used GFP (*dat-1p::gfp*) to track dopaminergic neuron loss over time, scoring the loss of ADE neuron fluorescence in the head region because ADE neurons are reported to be the most sensitive to α-synuclein-mediated neurodegeneration [10]. In addition, we found ADE neurons to be the easiest of the head dopaminergic neurons to visualize and therefore the most reliable to score. We observed no loss of ADE neurons in animals co-expressing GFP and untagged A30P α-synuclein at the age when we measured coiling (4 days post-hatching). For older adults (from day 7 onwards), a statistically significant proportion of A30P α-synuclein animals exhibited ADE neurodegeneration, compared to animals expressing GFP alone (Fig. 2a). Therefore, mild coiling of A30P α-synuclein animals is an early motor phenotype that precedes dopaminergic neuron loss.

To determine whether dopaminergic neuron dysfunction, in the absence of neuronal loss, could account for the motor impairment of A30P α-synuclein animals, we used a fluorescent reporter system that we previously characterized as an indicator of dopaminergic neuron signalling in *C. elegans* [45, 46]. In this system, *C. elegans* express a *cre::gfp* reporter gene in which the CRE (cAMP response element) DNA sequence is fused upstream of the gene encoding GFP [12]. CREB (CRE binding protein) is a transcription factor that, when activated, binds to the CRE sequence and induces transcription of genes downstream of CRE. In the CRE-GFP animal, GFP fluorescence is detected in cells in which activated CREB binds to CRE. Prior characterization of the CRE-GFP *C. elegans* demonstrated that, under normal conditions, GFP is seen in head mesodermal cells, some pharyngeal cells, and excretory glands. GFP is only rarely and weakly detected in neurons under normal conditions [12]. However, we previously discovered that GFP fluorescence can be induced in 4 cholinergic head neurons (named SIA) and regulated by dopamine (Fig. 2b) [45, 46]. SIA neurons receive synaptic input from the dopaminergic CEP neurons. We demonstrated that basal stimulation of the G-protein-coupled D2-like dopamine receptors expressed on SIA neurons inhibits CREB activation. This CREB inhibition due to upstream dopamine receptor stimulation is associated with minimal or no GFP expression in SIA neurons. Conversely, with reduced or absent dopaminergic neuron activity, stimulation of dopamine receptors on SIA neurons is limited, CREB is activated, and GFP expression is observed in these neurons [45, 46]. For example, the *cat-2(e1112)* animal with deficient dopaminergic neurotransmission due to mutation in the *cat-*2 gene, which encodes the enzyme tyrosine hydroxylase (TH) required for dopamine synthesis, exhibits frequent spontaneous GFP fluorescence in SIA neurons. Thus, minimal or absent GFP fluorescence in SIA neurons indicates intact dopaminergic neuron signalling, whereas spontaneous GFP fluorescence in SIA neurons indicates impairment of dopaminergic neuron signalling (Fig. 2b). Using this system, we tested whether the coiling phenotype of A30P α-synuclein animals is associated with impaired dopaminergic neuron signalling. Four-day-old young adult animals expressing A30P α-synuclein in dopaminergic neurons exhibited spontaneous GFP fluorescence in SIA neurons, similar to *cat-2(e1112)* animals (Fig. 2c). In contrast, control animals with the *cre::gfp* reporter gene, but without α-synuclein, displayed minimal spontaneous GFP signal in SIA neurons.

If coiling of A30P α-synuclein *C. elegans* occurs due to impaired dopaminergic neuron signaling, one predicts that activation of dopamine receptors with exogenous dopamine would reduce the phenotype. We examined the effect of dopamine treatment on motor behaviour and found that coiling of the animals decreased with treatment in a dose-dependent manner (Fig. 2d). Moreover, we induced impairment of dopaminergic neuron signaling in animals not expressing α-synuclein by treating them with raclopride, a D2 dopamine receptor antagonist. We found that D2 receptor blockade in these non-α-synuclein expressing animals recapitulated the coiling behaviour of transgenic α-synuclein *C. elegans* (Fig. 2d). The results from these pharmacological experiments imply that coiling can result from dopaminergic synaptic dysfunction, whether due to postsynaptic dopamine receptor antagonism or to impaired presynaptic signaling associated with α-synuclein expression.

Although coiling has been previously reported for several *unc C. elegans* strains, common molecular pathways for this motor phenotype have not been determined. To identify such pathways, we extracted strains that coil from a database of motor behaviours of 300 different *C. elegans* mutant strains (Supplementary Table 1) [16]. We then performed a gene ontology (GO) enrichment analysis of the genes mutated in the coiling strains to examine for the over-representation of genes associated with specific biological processes (Supplementary Table 2). Among the most highly over-represented gene classes associated with coiling were those related to dopamine receptor signaling, dopamine-related behaviour (i.e., response to food [46]), and dopamine metabolism (i.e., dopamine/catechol-containing metabolic/biosynthetic process) (Fig. 2e). These results taken together suggest that, prior to causing loss of dopamine neurons, α-synuclein accumulation in dopaminergic neurons leads to impaired presynaptic signalling, which results in the coiling behaviour.

### Alterations in protein control pathways that regulate α-synuclein accumulation affect coiling

**Fig. 3:**
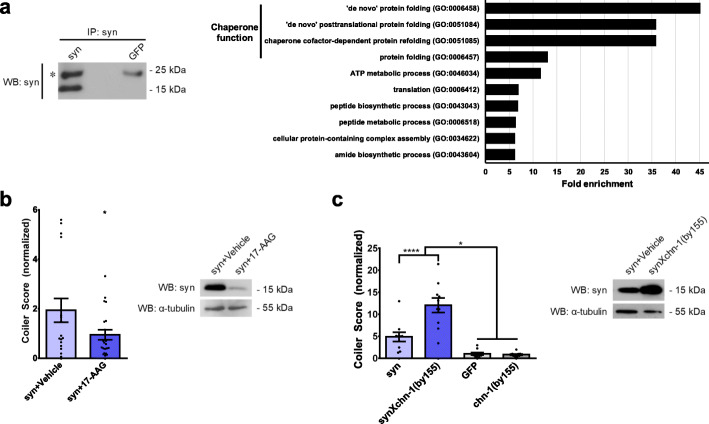
Alterations in protein control pathways that regulate α-synuclein accumulation affect coiling. (a) *Left:* Immunoblot of immunoprecipitated α-synuclein from syn *C. elegans* lysates. No α-synuclein was immunoprecipitated from GFP *C. elegans* lysates. The asterisk indicates the light chain of the immunoprecipitating antibody. Proteins co-immunoprecipitated with the anti-α-synuclein antibody were identified by mass spectrometry. *Right:* Proteins that were specifically co-immunoprecipitated from syn *C. elegans* lysates were analysed using gene ontology enrichment for biological process. Over-representation of proteins < 0.05 FDR was ranked according to fold enrichment. (b) *Left:* Coiler scores of syn animals treated with 50 μM 17-AAG (*N* = 21 populations of *n =* 10 animals) or vehicle (*N* = 17 populations of *n =* 10 animals), normalized to vehicle treated control GFP animals (two-tailed t-test, *p < 0.05). *Right*: Representative immunoblot for α-synuclein protein in lysates of treated animals (α-tubulin used as a loading control). (c) *Left:* Coiler scores of syn animals crossed with the mutant strain *chn-1(by155)*. CHN-1 is the homolog of the human co-chaperone protein, carboxyl-terminus of HSP70 interacting protein (CHIP). Coiler scores are normalized to control GFP animals (*N =* 10 populations of *n =* 10 animals for each strain) (one-way ANOVA with Tukey’s multiple comparisons test, *p < 0.05, ****p < 0.0001). *Right:* Representative immunoblot for α-synuclein protein in lysates of syn animals and syn animals crossed with *chn-1(by155)* (α-tubulin used as a loading control). Each data point represents an individual population

Abnormal accumulation of α-synuclein protein can occur in humans with PD due to defective cellular mechanisms responsible for protein quality control [47]. To determine which systems may be regulating α-synuclein accumulation in *C. elegans*, we identified proteins that bind to α-synuclein. Immunoprecipitations were performed from α-synuclein or GFP *C. elegans* lysates with anti-α-synuclein antibodies (Fig. 3a), and co-immunoprecipitated proteins were then identified by mass spectrometry (Supplementary Table 3). After excluding non-specific binding proteins that were present in both α-synuclein and GFP conditions, a total of 131 *C. elegans* proteins were identified as interacting with α-synuclein. These proteins were analysed using GO enrichment analysis (Supplementary Table 4). Among the α-synuclein interactors, we found that the most highly over-represented proteins were involved in chaperone function (Fig. 3a).

The chaperone system is composed of chaperone proteins, such as HSP70 (heat shock protein 70), and co-chaperones. Together, these proteins are integral to maintaining cellular protein quality control by folding newly translated polypeptides into their native conformation, refolding misfolded proteins to prevent their aggregation, and targeting proteins to degradation pathways, such as the autophagy-lysosomal system or ubiquitin-proteasome system, when refolding is unsuccessful. From the above in vitro findings, we predicted that perturbations to the chaperone system in vivo would affect coiling of the transgenic α-synuclein *C. elegans*. To test this prediction, we treated the animals with the geldanamycin analogue, 17-(allylamino)-17-demethoxygeldanamycin (17-AAG). Geldanamycin and its analogues are small molecules previously shown in cultured human cells to upregulate the chaperone system by increasing expression of HSP70 and to reduce α-synuclein accumulation and associated cell death [21, 48–50]. We found that 17-AAG treatment resulted in lower α-synuclein protein levels and reduced coiling by 51% compared to vehicle control (Fig. 3b). To examine the effect of downregulating the chaperone system, we crossed the transgenic α-synuclein *C. elegans* with the *chn-1(by155)* animal to generate an animal lacking functional CHIP (carboxyl-terminus of HSP70 interacting protein). CHIP is a co-chaperone of HSP70 that assists in refolding of proteins and directing proteins, such as α-synuclein, for degradation [18]. The *chn-1(by155)* animals without α-synuclein exhibited limited coiling, similar to the transgenic control GFP animals. In contrast, the *chn-1(by155)* animals with α-synuclein demonstrated a 47% increase in coiling, which was greater than the coiling behaviour of the α-synuclein alone animals (Fig. 3c). The more severe coiling was associated with higher levels of α-synuclein protein. Taken together, these results demonstrate that decreases or increases in α-synuclein *C. elegans* coiling occur with perturbations in protein quality control systems previously shown to inhibit or enhance cytotoxicity induced by α-synuclein, respectively. They also support our earlier observation that α-synuclein levels are positively correlated with coiling severity (Fig. 1f). Furthermore, these findings imply that a reduction in coiling of these animals may identify interventions, such as gene mutations or small molecules, that inhibit α-synuclein cytotoxicity in vivo.

### Application of the *C. elegans* model to a combined in silico, in vitro, and in vivo drug screen

**Fig. 4:**
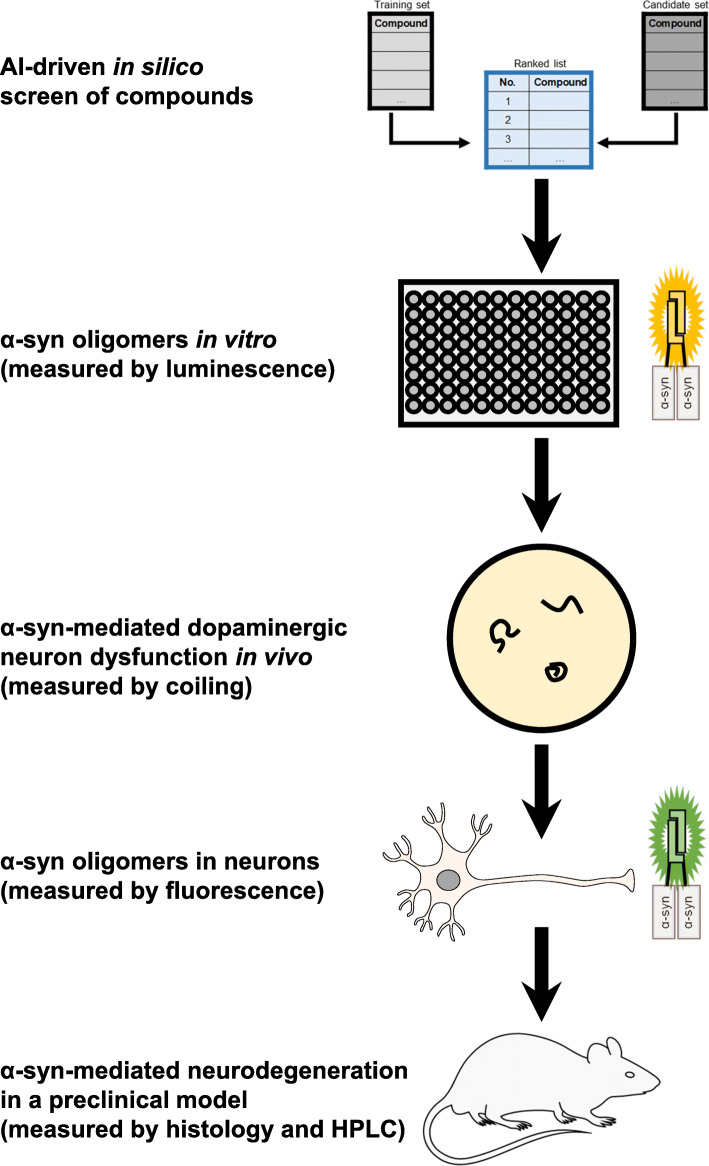
Screening strategy to identify compounds that inhibit α-synuclein oligomers and α-synuclein-mediated cytotoxicity. An AI-driven in silico screen was first performed with IBM Watson for Drug Discovery Predictive Analytics to predict compounds with a high likelihood of inhibiting aggregation of α-synuclein into oligomers. Next, highly ranked drugs were tested for their ability to reduce α-synuclein oligomer levels in vitro using a *Gaussia princeps* luciferase protein-fragment complementation cell assay. Positive hits were then assessed in vivo by measuring their effects on the motor impairment of *C. elegans* expressing α-synuclein. Last, screen hits were validated in mammalian in vitro and in vivo models. Specifically, drugs that reduced the coiling of *C. elegans* were examined for their ability to reduce α-synuclein oligomers measured by YFP protein-fragment complementation in rat primary cortical neurons, and our top candidate was tested in an AAV-based rat model of α-synuclein-mediated dopaminergic neurodegeneration

We explored the application of this *C. elegans* model by incorporating it into a screening strategy to identify compounds that inhibit α-synuclein aggregation and cytotoxicity (Fig. 4). In this strategy, we investigated drugs already approved for human use for treatment of diseases other than PD and thus have the potential for repurposing or repositioning [51]. We first used an AI-driven in silico method to predict compounds with a high likelihood of inhibiting aggregation of α-synuclein into oligomers. IBM Watson for Drug Discovery Predictive Analytics is a cloud-based computing platform that employs natural language processing to extract domain-specific text features from the abstracts of published manuscripts [52, 53]. It creates a semantic model from a set of known entities that share a common property of interest and then applies the model to rank a set of candidate entities according to their similarity to the known set using a graph diffusion algorithm [52, 53]. For our screen, the known entities were 15 small molecules previously shown to reduce α-synuclein oligomers in cell or animal models (Supplementary Table 5) [21, 48, 49, 54–60], and the candidate entities were 620 compounds currently prescribed for treatment of various human diseases and tracked in health care administrative databases of prescription drug utilization (Supplementary Table 6). We assessed the model’s performance with leave-one-out (LOO) cross-validation in which the graph diffusion algorithm was applied 15 times, each time with one entity removed from the known set and added to the candidate set. We found that 13 of the 15 known entities ranked in the top 30 when added to the 620 candidate entities, and the ranking scores of the known entities were significantly greater than those of the rest of the candidates (one-tailed Wilcoxon Sum Rank test, *p* = 7.02 × 10^− 10^) (Supplementary Table 6). The precision-recall curve had an average precision of 0.5 which was always above precision due to chance (percent positive = 0.02) (Supplementary Fig. 1a). In addition, the area under the Receiver-Operating Characteristic curve (AUC) was 0.975 (Supplementary Fig. 1b), further suggesting high predictive performance of the model (AUC = 1 corresponds to a perfectly predictive model). Using this model, the 620 candidate compounds were ranked by similarity to the known entities that reduce α-synuclein oligomers (Supplementary Table 6).

**Fig. 5:**
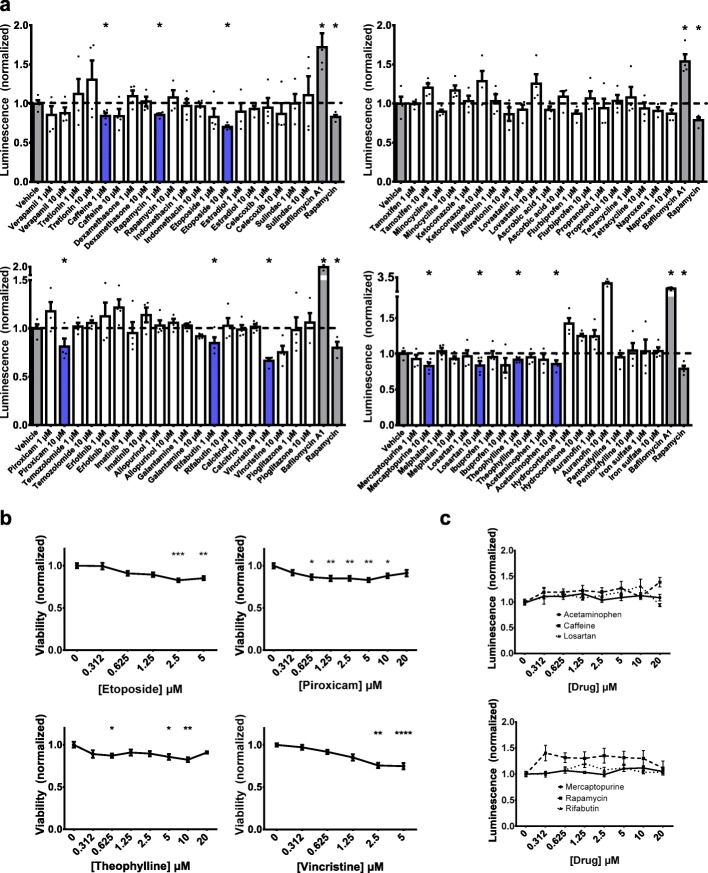
Acetaminophen, caffeine, losartan, mercaptopurine, rapamycin, and rifabutin reduce α-synuclein oligomers in vitro. (a) Testing of 40 highly ranked compounds (1 μM or 10 μM) for their ability to reduce α-synuclein oligomer levels in vitro using a *Gaussia* luciferase protein-fragment complementation cell assay. Rapamycin (2 μM) and bafilomycin A1 (50 nM) were used as active controls (*N =* 4 for each treatment) (one-tailed t-test, *p < 0.05). (b) Etoposide, piroxicam, theophylline, and vincristine demonstrated reductions in viability at multiple concentrations, measured using PrestoBlue-mediated fluorescence and normalized to vehicle alone (*N =* 3 with *n* = 2–4 replicates for each concentration) (one-way ANOVA with Dunnett’s post-hoc test, *p < 0.05, **p < 0.01, ***p < 0.001, ****p < 0.0001). No statistically significant changes in viability were observed with acetaminophen, caffeine, losartan, mercaptopurine, rapamycin, or rifabutin. (c) No statistically significant changes in the activity of full-length *Gaussia* luciferase alone were found with acetaminophen, caffeine, losartan, mercaptopurine, rapamycin, or rifabutin (*N =* 2 with *n* = 8 replicates for each concentration) (one-way ANOVA with Dunnett’s post-hoc test)

Next, 40 compounds ranked highly in silico based on predictions of their ability to reduce α-synuclein oligomers were tested in vitro (Supplementary Table 7). We used a bioluminescent protein-fragment complementation assay in which human H4 neuroglioma cells co-express human α-synuclein tagged with the N- or C-terminal half of *Gaussia princeps* luciferase (Fig. 4) [18, 21, 48, 49, 61]. Reconstitution of a complete and active luciferase molecule from the fragments occurs upon α-synuclein oligomerization and hence luminescence provides a surrogate measure of α-synuclein oligomer levels. For controls in this assay, we used vehicle (i.e., DMSO), rapamycin, and bafilomycin A1. Rapamycin, or sirolimus, is a small molecule that activates autophagy by inhibiting mTOR (mammalian target of rapamycin). Rapamycin and its derivatives have been found to inhibit neurodegeneration induced by α-synuclein in rodent models [62]. Bafilomycin A1 is an autophagy inhibitor previously shown to potentiate neurodegeneration due to α-synuclein in rodent models [63, 64]. In our assay, treatment of the cells with the autophagy inhibitor bafilomycin A1 resulted in an increase in α-synuclein oligomers, whereas treatment with rapamycin caused a decrease in α-synuclein oligomer levels (Fig. 5a). We identified 9 additional compounds as causing a statistically significant decrease in luciferase activity compared to vehicle alone: acetaminophen (14% decrease), caffeine (16% decrease), etoposide (30% decrease), losartan (16% decrease), mercaptopurine (17% decrease), piroxicam (19% decrease), rifabutin (15% decrease), theophylline (8% decrease), and vincristine (33% decrease) (Fig. 5a).

There are several potential causes for reduced luciferase activity in the bioluminescent protein-fragment complementation assay. First, it is possible that we would measure lower absolute α-synuclein oligomer levels if a compound caused cell death and thereby reduced the number of surviving α-synuclein-expressing cells. To test this possibility, we treated cells expressing α-synuclein with each of the compounds and used a cell permeable, resazurin-based assay to test for cell viability. We found that treatment with etoposide, piroxicam, theophylline, or vincristine, but not with the other compounds, reduced cell survival by > 15% (Fig. 5b). Thus, we excluded these four compounds from further study since their effects on α-synuclein oligomer measures may be secondary to cell death. Moreover, small molecules with significant cell toxicity are unlikely to be amenable to repurposing for a neurodegenerative disease. Second, it is also possible that we would measure lower luminescence if a compound acted directly on luciferase itself. We excluded this possibility by treating cells expressing only full-length luciferase. None of the remaining compounds (acetaminophen, caffeine, losartan, mercaptopurine, rapamycin, rifabutin) reduced the activity of luciferase alone (Fig. 5c, Supplementary Fig. 2). Third, luciferase activity might be reduced if a compound non-specifically lowered assembly of oligomer-prone proteins, such as α-synuclein, β-amyloid, and tau. We tested the effects of acetaminophen, caffeine, losartan, mercaptopurine, rapamycin, and rifabutin in bioluminescent protein-fragment complementation assays for β-amyloid and tau (Supplementary Fig. 3). One of the compounds reduced luminescence due to β-amyloid oligomers but this was associated with a comparable reduction in cell viability and thus confounded by cell death. None of the compounds lowered luminescence due to tau oligomers. Taken together, we inferred that acetaminophen, caffeine, losartan, mercaptopurine, rapamycin, and rifabutin each reduced the absolute amount of α-synuclein oligomers in vitro.

**Fig. 6:**
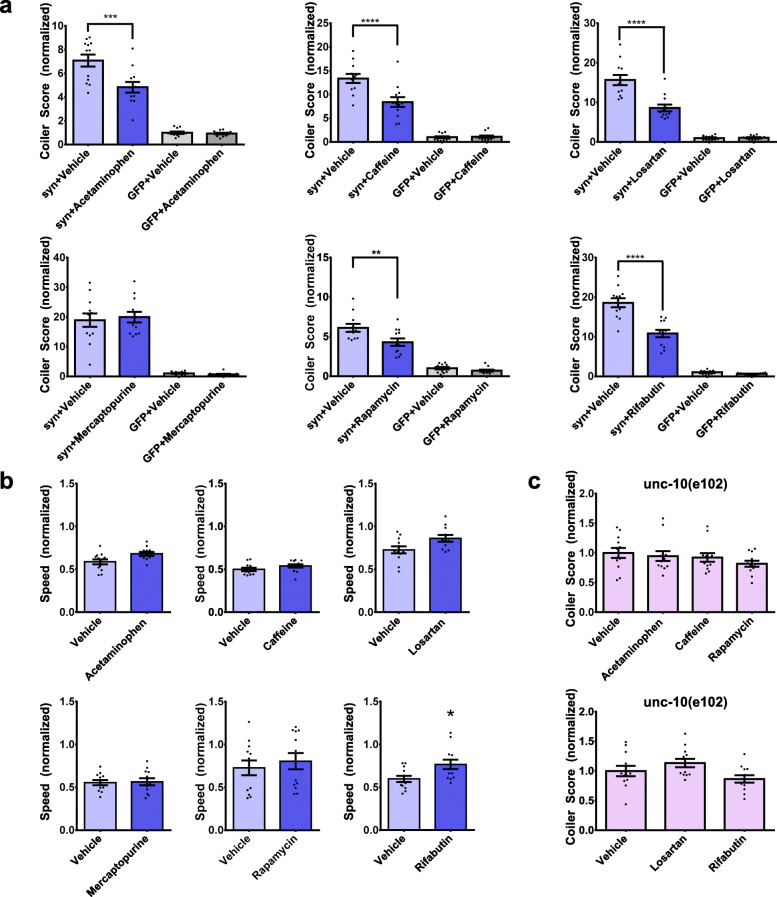
Acetaminophen, caffeine, losartan, rapamycin, and rifabutin reduce coiling due to α-synuclein-mediated dopaminergic neuron dysfunction in vivo. (a) Coiler scores of syn animals and GFP animals treated with vehicle or 100 μM of drug, normalized to vehicle treated control GFP animals (*N =* 12 populations of *n =* 10 animals per treatment group) (one-way ANOVA with Tukey’s post-hoc test, **p < 0.01, ***p < 0.001, ****p < 0.0001). (b) Locomotory speed of syn animals treated with vehicle or 100 μM of drug, normalized to vehicle treated control GFP animals (*N =* 12 populations of *n =* 10 animals per treatment group, two-tailed t-test, *p < 0.05). (c) No statistically significant changes in coiler scores of *unc-10(e102)* animals treated with vehicle or 100 μM of drug, normalized to vehicle treated *unc-10(e102)* animals (*N =* 12 populations of *n =* 10 animals per treatment group) (one-way ANOVA with Tukey’s post-hoc test). Each data point represents an individual population

We then applied our transgenic α-synuclein *C. elegans* model to test acetaminophen, caffeine, losartan, mercaptopurine, rapamycin, and rifabutin in vivo. We treated α-synuclein *C. elegans* or control GFP *C. elegans* with each compound or vehicle control. We found that treatment with 5 of the compounds caused a statistically significant reduction in coiling of α-synuclein *C. elegans* compared to vehicle alone: acetaminophen (32% decrease), caffeine (37% decrease), losartan (45% decrease), rapamycin (30% decrease), or rifabutin (42% decrease) (Fig. 6a). In contrast, mercaptopurine had no effect on the coiling behaviour of these animals. The minimal degree of coiling exhibited by control GFP *C. elegans* was unaffected by treatment with any of the compounds (Fig. 6a). We also measured locomotor speed since reduced speed due to treatment could be a confound by decreasing the frequency of coiling events. We found that treatment of these animals with each of the compounds did not decrease speed (Fig. 6b). To test whether the effects of acetaminophen, caffeine, losartan, rapamycin, and rifabutin were specific to coiling due to α-synuclein, we treated *unc-10(e102)* animals which display a similar degree of coiling as α-synuclein animals, but due to mutation in an ortholog of human RIMS1 (Fig. 1d) [34]. We found that none of these compounds affected the coiling behaviour of *unc-10(e102)* animals (Fig. 6c). Considering all the above data, we concluded that acetaminophen, caffeine, losartan, rapamycin, and rifabutin can each partially rescue the early motor impairment induced by α-synuclein in *C. elegans*.

### Validation of screen hits in mammalian models of α-synuclein-mediated neurodegeneration

**Fig. 7:**
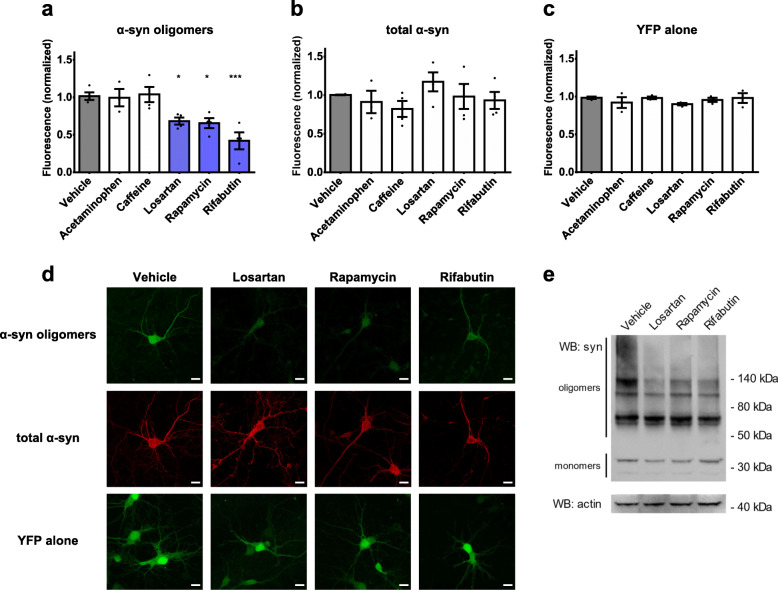
Losartan, rapamycin, and rifabutin reduce α-synuclein oligomers in mammalian neurons. (a) Five positive hits were tested for their ability to reduce α-synuclein oligomer levels in rat primary cortical neurons measured with YFP-based protein-fragment complementation. No statistically significant changes were observed in (b) total α-synuclein levels measured by immunohistochemistry or (c) fluorescence of full-length YFP alone. Fluorescence intensity was analyzed using Imaris image analysis software by measuring the mean fluorescence intensity of α-synuclein positive neurons throughout a 3D field of view generated by z-stack image acquisition using the “surfaces” module. The value generated for each neuron was normalized to the mean fluorescence intensity of neurons treated with vehicle alone (*N =* 3–4 replicates of *n =* 10 fields of view for each treatment; each data point represents an individual replicate) (nested one-way ANOVA with Dunnett’s post-hoc test, *p < 0.05, ***p < 0.001). (d) *Top & Middle:* Representative fluorescent microscopy images of neurons treated with vehicle, losartan, rapamycin, or rifabutin and co-expressing human α-synuclein tagged with the N- or C-terminal half of YFP. *Bottom:* Representative fluorescent microscopy images of neurons treated with vehicle, losartan, rapamycin, or rifabutin and expressing YFP alone. Scale bars are 10 μM. (e) Representative immunoblot to detect oligomers of split YFP-tagged α-synuclein (crosslinked) in rat primary cortical neuron lysates (actin used as a loading control)

Finally, we examined whether these 5 compounds that had effects in our invertebrate *C. elegans* model reduce α-synuclein oligomers and α-synuclein-mediated dopaminergic neurodegeneration in mammalian systems. Specifically, we used adeno-associated viral vectors (AAV) to co-express human α-synuclein tagged with the N- or C-terminal half of Venus yellow fluorescent protein (YFP) in rat cortical neurons in vitro. Similar to the luciferase protein-fragment complementation assay described above, reconstitution of a complete YFP molecule from the split YFP halves occurs upon α-synuclein oligomerization and thus spontaneous fluorescence provides an estimated measure of α-synuclein oligomer levels (Fig. 4) [21, 48, 49, 61, 65]. We treated these neurons with acetaminophen, caffeine, losartan, rapamycin, or rifabutin. We found that 3 compounds caused a statistically significant decrease in fluorescence compared to vehicle alone: losartan (33% decrease), rapamycin (36% decrease), and rifabutin (60% decrease) (Fig. 7a, d); this was associated with a reduction in α-synuclein oligomers as detected by immunoblotting (Fig. 7e). Total α-synuclein levels were not reduced in cultured neurons by any of the compounds when measured by immunofluorescence (Fig. 7b, d), but slight reductions in α-synuclein monomer levels by losartan, rapamycin, and rifabutin were detected by immunoblotting (Fig. 7e). In contrast, none of the compounds reduced YFP fluorescence when we treated neurons expressing full-length YFP alone (Fig. 7c, d). Thus, our screening strategy identified 3 compounds that reduce α-synuclein oligomer levels in mammalian neurons: rapamycin, which has previously demonstrated efficacy in an in vivo α-synuclein toxicity model of PD [62], losartan, which has previously shown neuroprotective potential but not via an α-synuclein-mediated mechanism [66, 67], and rifabutin, which has not yet been explored as a treatment for neurodegenerative diseases.

**Fig. 8:**
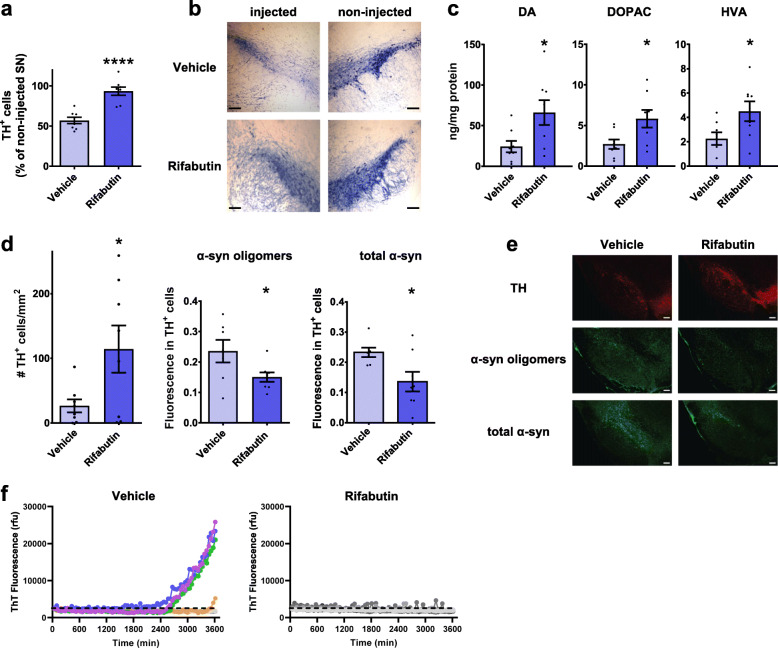
Rifabutin reduces α-synuclein oligomers and nigrostriatal dopaminergic neurodegeneration in an AAV-based α-synuclein rat model. (a) Dopaminergic neuron loss was induced by stereotactic injection of human A53T α-synuclein-expressing AAV in the unilateral SN. For vehicle treated animals, the number of dopaminergic (TH+) cells in the injected SN was only 57% of the non-injected SN versus 93% for rifabutin treated animals (*n =* 8 animals per treatment group, two-tailed t-test, ****p < 0.0001). (b) Representative images of TH staining of the SN injected with AAV-A53T α-synuclein and the contralateral non-injected SN from rats treated with vehicle or rifabutin. Scale bars are 200 μm. (c) Striatal levels of dopamine (DA) and its metabolites, DOPAC and HVA, were higher for rifabutin treated animals compared to vehicle treated animals (*n =* 8 animals per treatment group, two-tailed t-test, *p < 0.05). (d) Analyses of immunofluorescent images demonstrated that animals treated with rifabutin exhibited less dopaminergic (TH+) cell death in the injected SN compared to vehicle treated animals. Levels of α-synuclein oligomers and total human α-synuclein were lower in the surviving TH+ cells in the SN of rifabutin treated animals versus vehicle treated animals (*n* = 7–8 animals per treatment group, two-tailed t-test, *p < 0.05). (e) Representative images of immunofluorescent staining with anti-TH antibodies (to detect dopaminergic neurons), Syn-O2 (to detect α-synuclein oligomers), or Syn211 (to detect total human α-synuclein) in SN injected with AAV-A53T α-synuclein from rats treated with vehicle or rifabutin. Scale bars are 200 μm. (f) RT-QuIC spectra of CSF from 7 animals treated with vehicle and 8 animals treated with rifabutin. The dotted black line represents the threshold (mean background fluorescence + 5 SD) defining the positive and negative cases. Four vehicle treated cases were positive (coloured spectra) and 3 vehicle treated cases were negative (gray spectra). All 8 rifabutin treated cases were negative (gray spectra)

Rifabutin, a member of the ansamycin antibiotic family, is approved and currently used for chronic prophylactic treatment of disseminated *Mycobacterium avium* complex disease in people with HIV. To test the efficacy of rifabutin in reducing α-synuclein-medicated dopaminergic neurodegeneration in a mammalian model in vivo, we used an AAV-based rat model in which a unilateral stereotactic injection of virus results in expression of human mutant A53T α-synuclein in the dopaminergic neurons of the SN [29, 68] (the most prominently affected brain region in PD). Rats were randomized to receive vehicle or rifabutin at a dose equivalent to that used in humans [69]. Previous studies found no overt toxicological effects in rats at this dose [70]; consistent with this, we found no statistically significant differences in average body weights of animals receiving vehicle versus rifabutin over the course of treatment. The AAV-A53T model consistently shows loss of dopaminergic neurons in the injected SN, which we confirmed in this study using two separate methods. First, we demonstrated that the number of TH-positive cells in the AAV-A53T injected SN of vehicle treated animals was only 57% compared to the non-injected SN (Fig. 8a, b). Second, we showed that the number of TH-positive cells in the AAV-A53T injected SN of vehicle treated animals was significantly lower than the number of TH-positive cells in the SN of animals injected with an empty viral vector (EV) instead of AAV-A53T (Supplementary Fig. 4a, b). Although rifabutin is a relatively large molecule (847 Da), it is highly lipophilic and has been reported to demonstrate moderate penetration of the blood-brain barrier [71]. We confirmed brain delivery of rifabutin in our study by measuring steady-state drug concentrations in plasma and saline-perfused brain tissue from a subset of animals (Supplementary Fig. 4c,d). We found that animals treated with rifabutin demonstrated less dopaminergic cell death in the SN compared to those treated with vehicle (Fig. 8a, b, d and Supplementary Fig. 4a, b). This reduction in nigral neurodegeneration corresponded with higher levels of dopamine and its metabolites, 3,4-dihydroxyphenylacetic acid (DOPAC) and homovanillic acid (HVA), in the striatum (the primary brain region that receives projections from the SN dopaminergic neurons) (Fig. 8c). Furthermore, we found that the levels of α-synuclein oligomers and total α-synuclein in the surviving dopaminergic SN neurons were lowered with rifabutin treatment (Fig. 8d, e). To determine whether rifabutin reduced disease-associated α-synuclein conformations, we applied a real-time quaking induced conversion (RT-QuIC) assay to detect seeding activity of α-synuclein from CSF (Fig. 8f). Indeed, 0% of CSF samples from rifabutin treated animals demonstrated positive seeding activity, whereas CSF from 4 of the 7 vehicle treated animals (57%) tested positive by RT-QuIC (Fisher’s exact test, *p* = 0.03). These findings provide validation of the screening strategy used with our *C. elegans* model in mammalian in vitro and in vivo systems.

## Discussion

Here we have developed a simple in vivo model that recapitulates fundamental features of PD, including dopaminergic neuron dysfunction due to α-synuclein and motor impairment that improves with dopamine treatment. Since *C. elegans* are not known to express a homolog of α-synuclein, the neuronal dysfunction induced by expression of untagged α-synuclein and the correlation of motor dysfunction, specifically coiling, with α-synuclein levels suggests a toxic gain-of-function, which is a proposed mechanism of α-synuclein-mediated neurodegeneration in humans [72]. Coiling is a well described *unc* phenotype in *C. elegans* and our analysis of a database of *C. elegans* behavioural phenotypes [16] suggests a relationship to the dopamine system which has been underexplored until now. In our model, the coiler phenotype is caused by dopaminergic neuron dysfunction that precedes the loss of cell bodies and can be induced in non-α-synuclein expressing animals by pharmacological blockade of postsynaptic D2 receptors. Thus, we infer that coiling in this animal represents presynaptic dysfunction, which is a key feature of earlier stages of PD. [5]

It is at the earlier disease stages, when dopaminergic neuron cell bodies are intact and terminals are not completely degenerated, that treatment with disease-modifying therapies is expected to be most effective in PD. Yet, most models used for drug discovery do not have measurable and modifiable markers that reflect early α-synuclein-mediated neurodegeneration. Coiling in our model was not only an early phenotype but was also responsive to pharmacological or genetic manipulations that altered the chaperone system. These findings are relevant to PD in that chaperones are primary contributors to the maintenance of proteostasis and thus important regulators of aggregation prone proteins such as α-synuclein [47]. Further, our method of measuring the coiling behaviour assessed multiple animals simultaneously [73, 74], which could easily be scaled up and multiplexed, and thus there is the potential for developing a higher throughput system with this model.

We used this *C. elegans* model downstream of in silico and in vitro methods to identify compounds that inhibit neurodegeneration due to α-synuclein oligomers. Our in vitro methods used protein-fragment complementation systems previously shown to estimate the levels of α-synuclein oligomers associated with cell death (i.e., pathophysiological conformations of α-synuclein) in cell culture and in rodent models [21, 55, 65, 75–77]. The in silico methods, which incorporated AI components including natural language processing with machine learning [52, 53], were used for the first time for this purpose. Compared to phenotypic screening in cells alone, the addition of upstream in silico predictions appeared to increase the yield of positive hits (e.g., 3 out of a 620 compound library in our screen versus 2 out of a 1280 compound library in Moussaud et al. [49]). Thus, our results support the potential utility of combining AI technologies with in vitro and in vivo testing to facilitate drug discovery for PD.

The validity of our approach for discovering compounds with the potential to target α-synuclein is supported by one of the candidates we identified being rapamycin, a drug well known to enhance autophagy and thereby reduce α-synuclein accumulation [62]. Currently, targeting α-synuclein as a disease-modifying strategy for PD is an active area of drug development with small molecules, antibodies, and antisense oligonucleotides (ASOs) designed to reduce α-synuclein nearing or in human clinical trials [3]. We identified losartan, an angiotensin II receptor blocker, and rifabutin, an ansamycin antibiotic, as novel candidates which, unlike rapamycin, have not been previously explored as potential treatments to target α-synuclein-mediated neurodegeneration. Rifabutin is a particularly attractive candidate as a treatment for PD or other neurodegenerative diseases because it can penetrate the blood brain barrier. The compound library examined in this study only included drugs currently in use for treatment of various human diseases and here we demonstrate evidence of efficacy of rifabutin in a preclinical rodent model of PD and thus consideration could be made to rapidly transition to human clinical trials.

Limitations of our study include the possibility of false negatives with our drug screening approach due to the limited number of drug concentrations tested, the conservative cut-off used to exclude compounds for toxicity, and the potential absence of certain drug targets in the cell lines and animals used for testing. Further, while the simplicity of *C. elegans* makes it amenable to higher throughput analyses, it does not allow for all aspects of α-synuclein pathogenicity to be incorporated into a single model. Our model, for example, does not include a measure of cell-to-cell transmission of α-synuclein, which may contribute to the progression of neuropathology in PD. However, future studies could couple our model with other recently described *C. elegans* models that have indicators of cell-to-cell propagation to test compounds or pathways hypothesized to affect both dopaminergic dysfunction due to intracellular α-synuclein and its transmission [78, 79]. Similarly, the preclinical rodent model we used to test the efficacy of rifabutin recapitulates neurodegeneration due to intracellular α-synuclein but does not include a measure of α-synuclein spread. While our analyses of CSF from these animals suggest that rifabutin reduces extracellular α-synuclein, future work will require evaluation of the effects of rifabutin on the cellular release and uptake of α-synuclein as well as elucidation of the mechanism by which rifabutin reduces α-synuclein-mediated neurodegeneration.

## Conclusions

We identify for the first time the coiler phenotype as a *C. elegans* locomotor abnormality due to dopaminergic neuron dysfunction that models early α-synuclein-mediated neurodegeneration. Our validated approach applying this in vivo model to a multi-step drug repurposing screen with artificial intelligence-driven in silico and in vitro methods resulted in the discovery of at least one drug, rifabutin, that may now be investigated for repurposing as a disease-modifying therapy for Parkinson’s disease.

## Supplementary Information

**Additional file 1.**

## Data Availability

All data generated or analysed during this study are included or cited in this published article and its supplementary information files.

## Abbreviations

17-AAG: 17-(allylamino)-17-demethoxygeldanamycin
A30P: Alanine at amino acid position 30 mutated to proline
AAV: Adeno-associated viral vectors
ADE: Anterior deirid neuron
AI: Artificial intelligence
ASO: Antisense oligonucleotide
AUC: Area under the receiver-operating characteristic curve
CEP: Cephalic neuron
CHIP: Carboxyl-terminus of HSP70 interacting protein
CRE: cAMP response element
CREB: CRE binding protein
CSF: Cerebrospinal fluid
DMSO: Dimethyl sulfoxide
DOPAC: 3,4-dihyroxyphenylacetic acid
ELISA: Enzyme-linked immunosorbent assay
EV: Empty vector
FDR: False discovery rate
FUDR: Floxuridine
GFP: Green fluorescent protein
GO: Gene ontology
HIV: Human immunodeficiency virus
HPLC: High-performance liquid chromatography
HSP70: Heat shock protein 70
HVA: Homovanillic acid
LC-MS/MS: Liquid chromatography-tandem mass spectrometry
LOO: Leave-one-out cross-validation
MPP+: 1-methyl-4-phenylpyridinium
MPTP: 1-methyl-4-phenyl-1,2,3,6-tetrahydropyridine
mTOR: Mammalian target of rapamycin
NGM: Nematode growth medium
PD: Parkinson’s disease
PDE: Posterior deirid neuron
rfu: Relative fluorescence units
RIMS1: Regulating synaptic membrane exocytosis 1
ROC: Receiver-operating characteristic
ROI: Region of interest
RT-QuIC: Real-time quaking induced conversion
SCOCO: Short coiled-coil protein
SIA: Sublateral interneuron
SLC18A3: Solute carrier family 18 member A3
SN: Substantia nigra pars compacta
TH: Tyrosine hydroxylase
unc: Uncoordinated
YFP: Yellow fluorescent protein
