# Supplementary material for: Small molecule inhibitors of α-synuclein oligomers identified by targeting early dopamine-mediated motor impairment in C. elegans
Source: Mol Neurodegener. 2021 Nov 12;16:77. doi: 10.1186/s13024-021-00497-6 (PMC8588601; doi:10.1186/s13024-021-00497-6)
Supplement: Supplementary file 1 — Additional file 1. [file 13024_2021_497_MOESM1_ESM.pdf]

Supplementary Table 1. List of genes selected for gene ontology (GO) enrichment analysis based on genotypes reported to have a coiler phenotype in Yemini *et al.*[16] Genotypes were included if reported to demonstrate "time spent coiled" or "coil frequency" greater than control (q value ≤0.05). Genes are represented in alphabetical order (N=97).

Genes for GO Analysis	Genotypes with Coiler Phenotype
acd-2	*acd-2(ok1237)*
acd-5	*acd-5(ok2657)*
acr-3	*acr-3(ok2049)*
acr-9	*acr-9(ok933)*
asic-2	*asic-2(ok289)*
bas-1	*bas-1(ad446)*
cat-2	*cat-2(e1112)*
dnc-1	*dnc-1(or404)*
dop-1	*dop-1(vs100);dop-3(vs106)*
dop-2	*dop-2(vs105)*
dop-3	*dop-3(vs106)*
dop-4	*dop-4(tm1392)*
eat-16	*eat-16(sa609)*
egas-2	*egas-2(ok1477)*
egg-5	*egg-5(ok1781)*
egl-10	*egl-10(md176)*
egl-11	*egl-11(n587)*
egl-19	*egl-19(n2368)*
egl-2	*egl-2(n693)*
egl-20	*egl-20(mu39)*
egl-21	*egl-21(n611)*
egl-30	*egl-30(ep271)*
egl-9	*egl-9(n586)*
ets-10	*ets-10(gk596)*
flp-17	*flp-17(ok3587)*
flp-28	*flp-28(gk1075)*
gly-2	*gly-2(gk204)*
gpa-13	*gpa-13(pk1270)*
gpa-14	*gpa-14(pk347)*
gpa-15	*gpa-15(pk477)*
gpa-3	*gpa-3(pk35)*
gpa-5	*gpa-5(pk376)*
gpa-6	*gpa-6(pk480)*
hcf-1	*hcf-1(ok559)*
him-5	*spe-41(sy693); him-5(e1490)*
ins-25	*ins-25(ok2773)*
ins-28	*ins-28(ok2722)*
ins-3	*ins-3(ok2488)*
jnk-1	*jnk-1(gk7)*

lev-1	*lev-1(x427)*
lig-4	*lig-4(ok716)*
lon-2	*lon-2(e678)*
mec-10	*mec-10(u20)*
mec-12	*mec-12(e1605); bzIs17[pmec-4::YC2.12; lin-15(+)]*
mec-14	*mec-14(u55); bzIs18[pmec-4::YC2.12; lin-15(+)]*
mec-18	*mec-18(u228); bzIs17[pmec-4::YC2.12; lin-15(+)]*
mec-4	*mec-4(u253); bzIs17[pmec-4::YC2.12; lin-15(+)]*
mir-124	*mir-124(n4255)*
mod-1	*mod-1(ok103)*
nlp-1	*nlp-1(ok1469)*
nlp-17	*nlp-17(ok3461)*
npr-1	*npr-1(ad609)*
npr-12	*npr-12(tm1498)*
npr-2	*npr-2(ok419)*
npr-3	*npr-3(tm1583)*
ocr-4	*ocr-4(vs137)*
odr-3	*odr-3(n2150)*
rab-3	*rab-3(y250)*
ser-1	*ser-1(ok345)*
ser-2	*ser-2(pk1357)*
ser-5	*ser-5(tm2654)*
spe-41	*spe-41(sy693); him-5(e1490)*
syg-2	*syg-2(ky671)*
tbh-1	*tbh-1(n3247)*
tom-1	*tom-1(ok285)*
tph-1	*tph-1(mg280)*
trp-4	*trp-4(sy695)*
unc-1	*unc-1(e1598)*
unc-10	*unc-10(e102)*
unc-101	*unc-101(m1)*
unc-104	*unc-104(e1265)*
unc-105	*unc-105(ok1432)*
unc-108	*unc-108(n501)*
unc-115	*unc-115(mn481)*
unc-118	*unc-118(e2331)*
unc-122	*unc-122(e2520)*
unc-127	*unc-127(hs13)*
unc-18	*unc-18(e81)*
unc-2	*unc-2(gk366)*
unc-26	*unc-26(m2)*
unc-3	*unc-3(e151)*
unc-32	*unc-32(e189)*

unc-34	*unc-34(e566)*
unc-37	*unc-37(e262)*
unc-38	*unc-38(e264)*
unc-4	*unc-4(e120)*
unc-42	*unc-42(e270)*
unc-55	*unc-55(e402)*
unc-63	*unc-63(ok1075)*
unc-75	*unc-75(e950)*
unc-76	*unc-76(e911)*
unc-77	*unc-77(e625)*
unc-8	*unc-8(e15)*
unc-86	*unc-86(e1416)*
unc-9	*unc-9(e101)*
vab-7	*vab-7(e1562)*
zyg-9	*zyg-9(b244)*

Supplementary Table 2. Gene ontology (GO) enrichment analysis of *C. elegans* strains with coiler phenotype. Biological processes are listed if over-represented by ≥100-fold within the GO over-representation analysis of biological processes using PANTHER "GO biological process complete" (pantherdb.org/geneListAnalysis.do). *C. elegans* genome was used as the reference list. Biological processes with ≥100-fold enrichment were ranked according to $-\log_{10}$ of the false discovery rate (FDR).

Biological Process	Gene Ontology Term	# of Genes from Input	# of Genes Expected	$-\log_{10}$ (FDR)
Dopamine receptor signaling pathway	(GO:0007212)	8	0.07	11.266
Response to food	(GO:0032094)	5	0.04	6.669586
Calcium ion import across plasma membrane	(GO:0098703)	3	0.02	3.761954
Calcium ion import into cytosol	(GO:1902656)	3	0.02	3.759451
Adrenergic receptor signaling pathway	(GO:0071875)	3	0.02	3.756962
Adenylate cyclase-activating adrenergic receptor signaling pathway	(GO:0071880)	3	0.02	3.754487
Catechol-containing compound metabolic process	(GO:0009712)	3	0.03	3.605548
Catechol-containing compound biosynthetic process	(GO:0009713)	3	0.03	3.603801
Dopamine metabolic process	(GO:0042417)	3	0.03	3.60206
Catecholamine biosynthetic process	(GO:0042423)	3	0.03	3.598599
Catecholamine metabolic process	(GO:0006584)	3	0.03	3.596879
Synaptic target recognition	(GO:0008039)	2	0.01	2.31158
Serotonin biosynthetic process	(GO:0042427)	2	0.01	2.310691
Indole-containing compound biosynthetic process	(GO:0042435)	2	0.01	2.308919
Dopamine biosynthetic process from tyrosine	(GO:0006585)	2	0.01	2.307153
Primary amino compound biosynthetic process	(GO:1901162)	2	0.01	2.305395
Adenylate cyclase-activating dopamine receptor signaling pathway	(GO:0007191)	2	0.01	2.303644
Positive regulation of synaptic transmission, gabaergic	(GO:0032230)	2	0.02	2.155523
Regulation of synaptic vesicle exocytosis	(GO:2000300)	2	0.02	2.154282

Serotonin metabolic process	(GO:0042428)	2	0.02	2.152427
Nonassociative learning	(GO:0046958)	2	0.02	2.151195
Habituation	(GO:0046959)	2	0.02	2.149354
Primary amino compound metabolic process	(GO:1901160)	2	0.02	2.14813

Supplementary Table 3. All proteins co-immunoprecipitated with anti-α-synuclein antibodies from GFP or α-synuclein *C. elegans* lysates identified by mass spectrometry (N=207). Proteins identified in immunoprecipitants from α-synuclein *C. elegans* lysates but absent from GFP *C. elegans* lysates were selected for gene ontology enrichment analysis (N=131; highlighted in blue).

Identified Proteins	Accession Number	Total Spectrum Count	
		GFP	Syn
Uncharacterized protein OS=Caenorhabditis elegans GN=lev-11 PE=1 SV=1	tr|A0A0K3AUC2|A0A0K3AUC2_CAEEL	0	68
Myosin-4 OS=Caenorhabditis elegans GN=unc-54 PE=1 SV=2	sp|P02566|MYO4_CAEEL	0	33
Tropomyosin isoforms c/e OS=Caenorhabditis elegans GN=lev-11 PE=1 SV=1	sp|Q27249|TPM3_CAEEL	0	30
Nuclear anchorage protein 1 OS=Caenorhabditis elegans GN=anc-1 PE=1 SV=3	sp|Q9N4M4|ANC1_CAEEL	0	17
SaPosin-like Protein family OS=Caenorhabditis elegans GN=spp-10 PE=1 SV=3	tr|Q18276|Q18276_CAEEL (+1)	0	9
Uncharacterized protein OS=Caenorhabditis elegans GN=CELE_ZC247.1 PE=1 SV=1	tr|G5EBP5|G5EBP5_CAEEL	0	7
Uncharacterized protein OS=Caenorhabditis elegans GN=CELE_Y22D7AL.10 PE=1 SV=1	tr|Q965Q1|Q965Q1_CAEEL	0	7
Uncharacterized protein F13E6.1 OS=Caenorhabditis elegans GN=F13E6.1 PE=3 SV=2	sp|P55326|YZG1_CAEEL	0	7
Conserved Cysteine/Glycine domain protein OS=Caenorhabditis elegans GN=ccg-1 PE=1 SV=1	tr|Q9NA39|Q9NA39_CAEEL	0	7
ATP synthase subunit alpha, mitochondrial OS=Caenorhabditis elegans GN=H28O16.1 PE=1 SV=1	sp|Q9XXK1|ATPA_CAEEL	0	6
Prion-like-(Q/N-rich)-domain-bearing protein OS=Caenorhabditis elegans GN=pqn-22 PE=1 SV=2	tr|H2KZA3|H2KZA3_CAEEL (+9)	0	6
Cytochrome b-c1 complex subunit 6 OS=Caenorhabditis elegans GN=CELE_T27E9.2 PE=1 SV=1	tr|O45864|O45864_CAEEL	0	6
Probable malate dehydrogenase, mitochondrial OS=Caenorhabditis elegans GN=mdh-2 PE=3 SV=1	sp|O02640|MDHM_CAEEL	0	5
Fatty-acid and retinol-binding protein 2 OS=Caenorhabditis elegans GN=far-2 PE=1 SV=1	sp|P34383|FAR2_CAEEL	0	5
Uncharacterized protein OS=Caenorhabditis elegans GN=CELE_Y47G6A.15 PE=1 SV=1	tr|Q65XX4|Q65XX4_CAEEL	0	5
Uncharacterized protein OS=Caenorhabditis elegans GN=CELE_R09B3.3 PE=1 SV=1	tr|O45713|O45713_CAEEL	0	5
Protein Up-regulated in Daf-2(Gf)	tr|G5EBF3|G5EBF3_CAEEL	0	4

OS=Caenorhabditis elegans GN=pud-2.1 PE=1 SV=1			
GEX Interacting protein OS=Caenorhabditis elegans GN=gei-15 PE=1 SV=1	tr|G4SI07|G4SI07_CAEEL	0	4
Uncharacterized protein OS=Caenorhabditis elegans GN=CELE_H03A11.2 PE=1 SV=2	tr|G5EFE3|G5EFE3_CAEEL	0	4
Uncharacterized protein OS=Caenorhabditis elegans GN=osm-11 PE=1 SV=1	tr|O45346|O45346_CAEEL	0	4
Uncharacterized protein OS=Caenorhabditis elegans GN=CELE_E04F6.8 PE=1 SV=1	tr|Q19063|Q19063_CAEEL	0	4
Paramyosin OS=Caenorhabditis elegans GN=unc-15 PE=1 SV=1	sp|P10567|MYSP_CAEEL	0	3
Sorting NeXin OS=Caenorhabditis elegans GN=snx-13 PE=4 SV=1	tr|H1UBK0|H1UBK0_CAEEL (+2)	0	3
Superoxide dismutase [Cu-Zn] OS=Caenorhabditis elegans GN=sod-1 PE=1 SV=2	sp|P34697|SODC_CAEEL	0	3
Uncharacterized protein OS=Caenorhabditis elegans GN=CELE_M01H9.3 PE=1 SV=1	tr|H2L0G8|H2L0G8_CAEEL (+1)	0	3
Coiled coil Helix Coiled coiled Helix domain OS=Caenorhabditis elegans GN=chch-3 PE=1 SV=1	tr|Q21551|Q21551_CAEEL	0	3
Uncharacterized protein OS=Caenorhabditis elegans GN=C42D4.1 PE=1 SV=1	tr|Q18577|Q18577_CAEEL	0	3
RutC family protein C23G10.2 OS=Caenorhabditis elegans GN=C23G10.2 PE=3 SV=3	sp|Q10121|YSD2_CAEEL	0	3
Uncharacterized protein OS=Caenorhabditis elegans GN=CELE_Y43F8B.1 PE=1 SV=1	tr|B7FAR9|B7FAR9_CAEEL (+4)	0	3
Clathrin LIght Chain OS=Caenorhabditis elegans GN=clic-1 PE=1 SV=1	tr|P90961|P90961_CAEEL	0	3
Ribosomal protein, Large subunit, Acidic (P1) OS=Caenorhabditis elegans GN=rla-2 PE=1 SV=1	tr|Q9U1X9|Q9U1X9_CAEEL	0	3
Uncharacterized protein OS=Caenorhabditis elegans GN=CELE_R06C1.4 PE=1 SV=1	tr|O62337|O62337_CAEEL	0	3
GaLECtin OS=Caenorhabditis elegans GN=lec-2 PE=1 SV=2	tr|Q20684|Q20684_CAEEL	0	3
Ubiquitin-60S ribosomal protein L40 OS=Caenorhabditis elegans GN=ubq-2 PE=3 SV=2	sp|P49632|RL40_CAEEL	0	2
Heat shock 70 kDa protein A OS=Caenorhabditis elegans GN=hsp-1 PE=1 SV=2	sp|P09446|HSP7A_CAEEL	0	2
Glyceraldehyde-3-phosphate dehydrogenase 2 OS=Caenorhabditis elegans GN=gpd-2 PE=3 SV=2	sp|P17329|G3P2_CAEEL (+1)	0	2
Elongation factor 2 OS=Caenorhabditis elegans GN=eef-2 PE=2 SV=4	sp|P29691|EF2_CAEEL	0	2
Uncharacterized protein ZK637.2 OS=Caenorhabditis elegans GN=ZK637.2 PE=3 SV=2	sp|P30629|YOU2_CAEEL	0	2
Transthyretin-like protein 2 OS=Caenorhabditis elegans GN=ttr-2 PE=1 SV=1	sp|P34500|TTR2_CAEEL	0	2
ATP synthase subunit beta, mitochondrial	sp|P46561|ATPB_CAEEL	0	2

OS=Caenorhabditis elegans GN=atp-2 PE=1 SV=2			
Peptidyl-prolyl cis-trans isomerase 5 OS=Caenorhabditis elegans GN=cyn-5 PE=1 SV=2	sp|P52013|CYP5_CAEEL	0	2
Tubulin beta-2 chain OS=Caenorhabditis elegans GN=tbb-2 PE=3 SV=1	sp|P52275|TBB2_CAEEL (+2)	0	2
Small ubiquitin-related modifier OS=Caenorhabditis elegans GN=smo-1 PE=1 SV=1	sp|P55853|SUMO_CAEEL	0	2
Tubulin polymerization-promoting protein homolog OS=Caenorhabditis elegans GN=tppp-1 PE=1 SV=1	sp|P91127|TPPP_CAEEL	0	2
Uncharacterized protein T28D9.1 OS=Caenorhabditis elegans GN=T28D9.1 PE=4 SV=2	sp|Q10020|YSX1_CAEEL	0	2
NHP2-like protein 1 homolog OS=Caenorhabditis elegans GN=M28.5 PE=3 SV=1	sp|Q21568|NH2L1_CAEEL	0	2
Probable voltage-dependent anion-selective channel OS=Caenorhabditis elegans GN=vdac-1 PE=3 SV=2	sp|Q21752|VDAC_CAEEL	0	2
Myosin-2 essential light chain OS=Caenorhabditis elegans GN=mlc-5 PE=1 SV=1	sp|Q9XVI9|MLC5_CAEEL	0	2
PeRoxireDoXin OS=Caenorhabditis elegans GN=prdx-2 PE=1 SV=1	tr|A0A0K3AUJ9|A0A0K3AUJ9_CAEEL (+2)	0	2
MLP/CRP family (Muscle LIM Protein/Cysteine-rich Protein) OS=Caenorhabditis elegans GN=mlp-1 PE=1 SV=1	tr|H2KZV8|H2KZV8_CAEEL (+1)	0	2
Uncharacterized protein OS=Caenorhabditis elegans GN=C49G7.3 PE=1 SV=1	tr|O16226|O16226_CAEEL	0	2
PHAryngeal gland Toxin-related OS=Caenorhabditis elegans GN=phat-4 PE=1 SV=1	tr|O16424|O16424_CAEEL	0	2
Heat Shock Protein OS=Caenorhabditis elegans GN=hsp-25 PE=1 SV=1	tr|Q17849|Q17849_CAEEL (+1)	0	2
Uncharacterized protein OS=Caenorhabditis elegans GN=C15C7.5 PE=1 SV=1	tr|Q18012|Q18012_CAEEL	0	2
Uncharacterized protein OS=Caenorhabditis elegans GN=C15H9.9 PE=1 SV=1	tr|Q18032|Q18032_CAEEL	0	2
Uncharacterized protein OS=Caenorhabditis elegans GN=CELE_T28F4.5 PE=1 SV=1	tr|Q22850|Q22850_CAEEL	0	2
Ribosomal Protein, Small subunit OS=Caenorhabditis elegans GN=rps-20 PE=1 SV=1	tr|Q8WQA8|Q8WQA8_CAEEL	0	2
CathePsin L family OS=Caenorhabditis elegans GN=cpl-1 PE=1 SV=1	tr|O45734|O45734_CAEEL	0	2
SaPosin-like Protein family OS=Caenorhabditis elegans GN=spp-15 PE=4 SV=1	tr|Q9BIC4|Q9BIC4_CAEEL	0	2
Myosin-3 OS=Caenorhabditis elegans GN=myo-3 PE=2 SV=1	sp|P12844|MYO3_CAEEL	0	2
Heat shock protein Hsp-12.2 OS=Caenorhabditis elegans GN=hsp-12.2	sp|P34328|HSP10_CAEEL	0	2

PE=3 SV=1			
Histone H4 OS=Caenorhabditis elegans GN=his-1 PE=1 SV=2	sp|P62784|H4_CAEEL	0	2
Uncharacterized protein OS=Caenorhabditis elegans GN=C53B7.3 PE=1 SV=1	tr|H2KZE2|H2KZE2_CAEEL (+2)	0	2
Uncharacterized protein OS=Caenorhabditis elegans GN=CELE_Y53G8AR.6 PE=1 SV=1	tr|Q9N3G0|Q9N3G0_CAEEL	0	2
Histone H2A.V OS=Caenorhabditis elegans GN=htz-1 PE=2 SV=3	sp|Q27511|H2AV_CAEEL	0	2
Chondroitin proteoglycan 1 OS=Caenorhabditis elegans GN=cpg-1 PE=1 SV=1	sp|Q17802|CPG1_CAEEL	0	1
40S ribosomal protein S17 OS=Caenorhabditis elegans GN=rps-17 PE=3 SV=2	sp|O01692|RS17_CAEEL	0	1
Neuropeptide-like protein 31 OS=Caenorhabditis elegans GN=nlp-31 PE=2 SV=1	sp|O44662|NLP31_CAEEL (+7)	0	1
Papilin OS=Caenorhabditis elegans GN=mig-6 PE=1 SV=1	sp|O76840|PPN1_CAEEL	0	1
Myosin-2 OS=Caenorhabditis elegans GN=myo-2 PE=1 SV=2	sp|P12845|MYO2_CAEEL	0	1
Cuticle collagen 8 OS=Caenorhabditis elegans GN=col-8 PE=3 SV=2	sp|P18833|COL8_CAEEL	0	1
Tubulin alpha-2 chain OS=Caenorhabditis elegans GN=tba-2 PE=2 SV=1	sp|P34690|TBA2_CAEEL (+5)	0	1
14-3-3-like protein 1 OS=Caenorhabditis elegans GN=par-5 PE=1 SV=2	sp|P41932|14331_CAEEL	0	1
Chaperonin homolog Hsp-60, mitochondrial OS=Caenorhabditis elegans GN=hsp-60 PE=2 SV=2	sp|P50140|CH60_CAEEL (+2)	0	1
NADH dehydrogenase [ubiquinone] 1 beta subcomplex subunit 7 OS=Caenorhabditis elegans GN=D2030.4 PE=3 SV=1	sp|P90789|NDUB7_CAEEL	0	1
Probable V-type proton ATPase subunit G OS=Caenorhabditis elegans GN=vha-10 PE=3 SV=1	sp|P91303|VATG_CAEEL	0	1
Transthyretin-like protein 5 OS=Caenorhabditis elegans GN=ttr-5 PE=3 SV=1	sp|Q03575|TTR5_CAEEL	0	1
Uncharacterized protein F15G9.1 OS=Caenorhabditis elegans GN=F15G9.1 PE=4 SV=1	sp|Q10033|YSI1_CAEEL (+1)	0	1
Probable arginine kinase F46H5.3 OS=Caenorhabditis elegans GN=F46H5.3 PE=3 SV=2	sp|Q10454|KARG1_CAEEL	0	1
Aminopeptidase-like protein AC3.5 OS=Caenorhabditis elegans GN=AC3.5 PE=1 SV=2	sp|Q17405|YQ02_CAEEL (+1)	0	1
Probable arginine--tRNA ligase, cytoplasmic OS=Caenorhabditis elegans GN=rrt-1 PE=3 SV=2	sp|Q19825|SYRC_CAEEL (+2)	0	1
Translation machinery-associated protein 7 homolog OS=Caenorhabditis elegans GN=F49C12.11 PE=3 SV=1	sp|Q20588|TMA7_CAEEL	0	1

Sperm-specific class P protein 9/11 OS=Caenorhabditis elegans GN=ssp-9 PE=2 SV=1	sp|Q23058|SSP9_CAEEL	0	1
Uncharacterized protein D2005.3 OS=Caenorhabditis elegans GN=D2005.3 PE=3 SV=2	sp|Q93408|YRGK_CAEEL	0	1
60S ribosomal protein L10a OS=Caenorhabditis elegans GN=rpl-10a PE=3 SV=1	sp|Q9N4I4|RL10A_CAEEL	0	1
Probable splicing factor, arginine/serine-rich 3 OS=Caenorhabditis elegans GN=rsp-3 PE=1 SV=2	sp|Q9NEW6|RSP3_CAEEL	0	1
40S ribosomal protein S27 OS=Caenorhabditis elegans GN=rps-27 PE=1 SV=3	sp|Q9TXP0|RS27_CAEEL	0	1
Mitochondrial import inner membrane translocase subunit Tim10 OS=Caenorhabditis elegans GN=tin-10 PE=3 SV=1	sp|Q9Y0V6|TIM10_CAEEL	0	1
Synaptosomal-associated protein OS=Caenorhabditis elegans GN=ric-4 PE=1 SV=1	tr|A5PEW5|A5PEW5_CAEEL (+1)	0	1
Uncharacterized protein OS=Caenorhabditis elegans GN=C49G7.7 PE=4 SV=1	tr|B1GRK2|B1GRK2_CAEEL	0	1
Oxysterol-binding protein OS=Caenorhabditis elegans GN=obr-2 PE=1 SV=1	tr|B2D6P3|B2D6P3_CAEEL (+2)	0	1
Glycine Rich Secreted Protein OS=Caenorhabditis elegans GN=grsp-2 PE=1 SV=1	tr|C6KRI7|C6KRI7_CAEEL (+6)	0	1
Cystatin OS=Caenorhabditis elegans GN=cpi-1 PE=1 SV=1	tr|G5EDZ9|G5EDZ9_CAEEL	0	1
Hsp-70 Interacting Protein homolog OS=Caenorhabditis elegans GN=hip-1 PE=1 SV=1	tr|G5EE04|G5EE04_CAEEL	0	1
CU (Copper) Chaperonin OS=Caenorhabditis elegans GN=cuc-1 PE=1 SV=1	tr|G5EE41|G5EE41_CAEEL	0	1
Major Sperm protein Domain containing OS=Caenorhabditis elegans GN=msd-2 PE=1 SV=1	tr|G5EEJ7|G5EEJ7_CAEEL	0	1
COLlagen OS=Caenorhabditis elegans GN=col-140 PE=1 SV=1	tr|H2KYE5|H2KYE5_CAEEL (+1)	0	1
Uncharacterized protein OS=Caenorhabditis elegans GN=CELE_R05A10.1 PE=1 SV=1	tr|H2L2I9|H2L2I9_CAEEL (+1)	0	1
Dauer Up-Regulated OS=Caenorhabditis elegans GN=dur-1 PE=1 SV=1	tr|H9G2T6|H9G2T6_CAEEL (+5)	0	1
COLlagen OS=Caenorhabditis elegans GN=col-184 PE=1 SV=1	tr|I2HA94|I2HA94_CAEEL (+1)	0	1
TropoNin T OS=Caenorhabditis elegans GN=tnt-4 PE=1 SV=1	tr|K8ERV5|K8ERV5_CAEEL (+1)	0	1
Acyl CoA DeHydrogenase OS=Caenorhabditis elegans GN=acdh-5 PE=1 SV=1	tr|O01502|O01502_CAEEL	0	1
GRounDhog (Hedgehog-like family) OS=Caenorhabditis elegans GN=grd-14	tr|O02153|O02153_CAEEL (+1)	0	1

PE=1 SV=2			
GRounDhog (Hedgehog-like family) OS=Caenorhabditis elegans GN=grd-5 PE=1 SV=1	tr|O16462|O16462_CAEEL	0	1
Ribosomal Protein, Small subunit OS=Caenorhabditis elegans GN=rps-18 PE=1 SV=1	tr|O18240|O18240_CAEEL	0	1
PERMeable eggshell OS=Caenorhabditis elegans GN=perm-2 PE=1 SV=2	tr|O44145|O44145_CAEEL	0	1
GRounDhog (Hedgehog-like family) OS=Caenorhabditis elegans GN=grd-10 PE=1 SV=1	tr|O44471|O44471_CAEEL	0	1
LAMinin related. See also lmb OS=Caenorhabditis elegans GN=lam-1 PE=1 SV=3	tr|O44565|O44565_CAEEL	0	1
C-type LECtin OS=Caenorhabditis elegans GN=clec-63 PE=1 SV=1	tr|O45444|O45444_CAEEL	0	1
Uncharacterized protein OS=Caenorhabditis elegans GN=CELE_F45H10.2 PE=1 SV=1	tr|O45525|O45525_CAEEL (+1)	0	1
Uncharacterized protein OS=Caenorhabditis elegans GN=CELE_T12D8.5 PE=1 SV=2	tr|O45783|O45783_CAEEL	0	1
TransThyretin-Related family domain OS=Caenorhabditis elegans GN=ttr-51 PE=1 SV=2	tr|O62289|O62289_CAEEL	0	1
COLlagen OS=Caenorhabditis elegans GN=col-73 PE=1 SV=1	tr|P91250|P91250_CAEEL	0	1
Uncharacterized protein OS=Caenorhabditis elegans GN=unc-44 PE=1 SV=2	tr|Q17489|Q17489_CAEEL	0	1
FUS/TLS RNA binding protein homolog OS=Caenorhabditis elegans GN=fust-1 PE=1 SV=2	tr|Q18265|Q18265_CAEEL	0	1
Related to yeast Vacuolar Protein Sorting factor OS=Caenorhabditis elegans GN=vps-32.1 PE=1 SV=1	tr|Q18886|Q18886_CAEEL	0	1
Uncharacterized protein OS=Caenorhabditis elegans GN=CELE_F52A8.1 PE=1 SV=1	tr|Q20637|Q20637_CAEEL	0	1
Uncharacterized protein OS=Caenorhabditis elegans GN=CELE_K02F3.9 PE=1 SV=1	tr|Q21151|Q21151_CAEEL	0	1
Sperm-Specific family, class Q OS=Caenorhabditis elegans GN=ssq-1 PE=1 SV=1	tr|Q21294|Q21294_CAEEL	0	1
Peptidyl-prolyl cis-trans isomerase OS=Caenorhabditis elegans GN=FKBP-2 PE=2 SV=1	tr|Q27462|Q27462_CAEEL (+1)	0	1
Dauer or Aging adult Overexpression OS=Caenorhabditis elegans GN=dao-2 PE=1 SV=1	tr|Q86LS4|Q86LS4_CAEEL	0	1
TransThyretin-Related family domain OS=Caenorhabditis elegans GN=ttr-41 PE=1 SV=1	tr|Q86NH9|Q86NH9_CAEEL	0	1
Uncharacterized protein OS=Caenorhabditis elegans GN=CELE_F56C9.11 PE=4 SV=2	tr|Q8WQE8|Q8WQE8_CAEEL	0	1
Nucleoside diphosphate kinase OS=Caenorhabditis elegans GN=ndk-1 PE=1 SV=1	tr|Q93576|Q93576_CAEEL	0	1

Vacuolar H ATPase OS=Caenorhabditis elegans GN=vha-8 PE=1 SV=1	tr|Q95X44|Q95X44_CAEEL	0	1
TuBulin folding Cofactor A homolog OS=Caenorhabditis elegans GN=tbca-1 PE=1 SV=1	tr|Q95XR1|Q95XR1_CAEEL	0	1
Uncharacterized protein OS=Caenorhabditis elegans GN=CELE_Y22D7AR.10 PE=1 SV=1	tr|Q9BKX4|Q9BKX4_CAEEL	0	1
GLutaRedoXin OS=Caenorhabditis elegans GN=glrx-10 PE=1 SV=1	tr|Q9N456|Q9N456_CAEEL	0	1
Uncharacterized protein OS=Caenorhabditis elegans GN=CELE_H20J04.1 PE=4 SV=1	tr|Q9N5L7|Q9N5L7_CAEEL	0	1
MBF (Multiprotein bridging factor) transcriptional coactivator OS=Caenorhabditis elegans GN=mbf-1 PE=1 SV=1	tr|Q9XTV4|Q9XTV4_CAEEL	0	1
Uncharacterized protein OS=Caenorhabditis elegans GN=C06C6.6 PE=4 SV=1	tr|O62032|O62032_CAEEL	0	1
Fatty-acid and retinol-binding protein 1 OS=Caenorhabditis elegans GN=far-1 PE=3 SV=1	sp|P34382|FAR1_CAEEL	1	18
Uncharacterized protein OS=Caenorhabditis elegans GN=CELE_K08D12.3 PE=1 SV=1	tr|Q966I7|Q966I7_CAEEL	1	7
G Protein, Gamma subunit OS=Caenorhabditis elegans GN=gpc-2 PE=1 SV=1	tr|G5ECT6|G5ECT6_CAEEL	1	6
Uncharacterized protein OS=Caenorhabditis elegans GN=CELE_ZC373.2 PE=1 SV=2	tr|Q23258|Q23258_CAEEL	1	5
Uncharacterized protein OS=Caenorhabditis elegans GN=CELE_Y44A6D.2 PE=1 SV=1	tr|Q9XXE2|Q9XXE2_CAEEL	1	5
CALUmenin (Calcium-binding protein) homolog OS=Caenorhabditis elegans GN=calu-1 PE=1 SV=1	tr|G5EBH7|G5EBH7_CAEEL	1	4
Histone H2A OS=Caenorhabditis elegans GN=his-3 PE=1 SV=2	sp|P09588|H2A_CAEEL (+3)	1	4
Elongation factor 1-alpha OS=Caenorhabditis elegans GN=eft-3 PE=3 SV=1	sp|P53013|EF1A_CAEEL (+1)	1	4
ATP synthase subunit delta, mitochondrial OS=Caenorhabditis elegans GN=F58F12.1 PE=1 SV=1	sp|Q09544|ATPD_CAEEL	1	3
Mitochondrial import inner membrane translocase subunit tim-13 OS=Caenorhabditis elegans GN=tin-13 PE=3 SV=1	sp|O45319|TIM13_CAEEL	1	3
Ferritin OS=Caenorhabditis elegans GN=ftn-2 PE=1 SV=1	tr|Q9TYS3|Q9TYS3_CAEEL	1	3
Cytochrome c 2.1 OS=Caenorhabditis elegans GN=cyc-2.1 PE=1 SV=2	sp|P19974|CYC21_CAEEL	1	3
Cytochrome b-c1 complex subunit Rieske, mitochondrial OS=Caenorhabditis elegans GN=isp-1 PE=1 SV=1	tr|O44512|O44512_CAEEL	1	2
Calponin Homology Domain containing Protein OS=Caenorhabditis elegans GN=chdp-1 PE=1 SV=1	tr|P91027|P91027_CAEEL	1	2
Basement membrane proteoglycan	sp|Q06561|UNC52_CAEEL (+8)	1	2

OS=Caenorhabditis elegans GN=unc-52 PE=1 SV=2			
Adenosine Deaminase acting on RNA OS=Caenorhabditis elegans GN=adr-1 PE=1 SV=1	tr|Q86GC2|Q86GC2_CAEEL (+5)	1	2
Aspartic protease 6 OS=Caenorhabditis elegans GN=asp-6 PE=3 SV=1	sp|O01530|ASP6_CAEEL	1	2
Probable prefoldin subunit 4 OS=Caenorhabditis elegans GN=pfd-4 PE=2 SV=1	sp|Q17435|PFD4_CAEEL	1	2
Mitochondrial import inner membrane translocase subunit tim-8 OS=Caenorhabditis elegans GN=tim-8 PE=3 SV=1	sp|Q9N408|TIM8_CAEEL	1	2
Uncharacterized protein OS=Caenorhabditis elegans GN=oxy-5 PE=1 SV=1	tr|A7DT45|A7DT45_CAEEL	1	2
Uncharacterized protein OS=Caenorhabditis elegans GN=CELE_W05H9.1 PE=1 SV=1	tr|Q23179|Q23179_CAEEL	1	2
Uncharacterized protein OS=Caenorhabditis elegans GN=C45B2.1 PE=1 SV=1	tr|Q95YC6|Q95YC6_CAEEL	1	2
LiPid Depleted OS=Caenorhabditis elegans GN=lpd-5 PE=1 SV=2	tr|Q9N4L8|Q9N4L8_CAEEL	1	2
Neuropeptide-Like Protein OS=Caenorhabditis elegans GN=nlp-40 PE=1 SV=1	tr|Q9N4D8|Q9N4D8_CAEEL (+1)	1	2
Uncharacterized protein OS=Caenorhabditis elegans GN=CELE_D1086.10 PE=1 SV=1	tr|B1Q273|B1Q273_CAEEL (+1)	1	1
PERMeable eggshell OS=Caenorhabditis elegans GN=perm-4 PE=1 SV=2	tr|O44144|O44144_CAEEL	1	1
C. Elegans Y-box OS=Caenorhabditis elegans GN=cey-1 PE=1 SV=1	tr|O62213|O62213_CAEEL	1	1
Glycine Rich Secreted Protein OS=Caenorhabditis elegans GN=grsp-4 PE=1 SV=1	tr|P91207|P91207_CAEEL	1	1
Homologous to Drosophila SQD (Squid) protein OS=Caenorhabditis elegans GN=sqd-1 PE=1 SV=1	tr|Q4W5P0|Q4W5P0_CAEEL (+1)	1	1
Muscle M-line assembly protein unc-89 OS=Caenorhabditis elegans GN=unc-89 PE=1 SV=3	sp|O01761|UNC89_CAEEL	1	0
Uncharacterized protein ZK632.9 OS=Caenorhabditis elegans GN=ZK632.9 PE=4 SV=3	sp|P34654|YOT9_CAEEL	1	0
Probable small nuclear ribonucleoprotein G OS=Caenorhabditis elegans GN=snr-7 PE=3 SV=1	sp|Q9N4G9|RUXG_CAEEL	1	0
Uncharacterized protein OS=Caenorhabditis elegans GN=CELE_F44E5.4 PE=1 SV=1	tr|G5ECU5|G5ECU5_CAEEL (+1)	1	0
Uncharacterized protein OS=Caenorhabditis elegans GN=CELE_T28C6.7 PE=1 SV=5	tr|Q22847|Q22847_CAEEL (+1)	1	0
Heat Shock factor Binding protein OS=Caenorhabditis elegans GN=hsb-1 PE=1 SV=1	tr|Q9U3B7|Q9U3B7_CAEEL	1	0
Actin-3 OS=Caenorhabditis elegans GN=act-3 PE=1 SV=1	sp|P0DM42|ACT3_CAEEL (+4)	2	10
Uncharacterized protein OS=Caenorhabditis	tr|O02267|O02267_CAEEL	2	7

elegans GN=CELE_F45H10.3 PE=1 SV=1			
SaPosin-like Protein family OS=Caenorhabditis elegans GN=spp-5 PE=1 SV=1	tr|Q86FL8|Q86FL8_CAEEL	2	6
SaPosin-like Protein family OS=Caenorhabditis elegans GN=spp-2 PE=1 SV=1	tr|Q8MPX7|Q8MPX7_CAEEL	2	6
ASpartyl Protease OS=Caenorhabditis elegans GN=asp-1 PE=1 SV=1	tr|G5EEI4|G5EEI4_CAEEL (+1)	2	5
Nematode Polyprotein Allergen related OS=Caenorhabditis elegans GN=npa-1 PE=1 SV=1	tr|G5EF32|G5EF32_CAEEL	2	4
60S acidic ribosomal protein P1 OS=Caenorhabditis elegans GN=rla-1 PE=3 SV=2	sp|P91913|RLA1_CAEEL	2	4
Uncharacterized protein OS=Caenorhabditis elegans GN=CELE_F42A10.5 PE=1 SV=1	tr|Q20310|Q20310_CAEEL	2	3
Uncharacterized protein OS=Caenorhabditis elegans GN=CELE_T24B8.3 PE=1 SV=2	tr|Q22719|Q22719_CAEEL (+1)	2	2
RNA-binding protein 8A OS=Caenorhabditis elegans GN=rnp-4 PE=1 SV=1	tr|Q21832|Q21832_CAEEL	2	0
UPF0375 protein C08F11.11 OS=Caenorhabditis elegans GN=C08F11.11 PE=1 SV=1	sp|O62053|U375A_CAEEL	3	8
40S ribosomal protein S28 OS=Caenorhabditis elegans GN=rps-28 PE=3 SV=1	sp|Q95Y04|RS28_CAEEL	3	6
FIP (Fungus-Induced Protein) Related OS=Caenorhabditis elegans GN=fipr-21 PE=1 SV=1	tr|Q20277|Q20277_CAEEL	3	6
Uncharacterized protein OS=Caenorhabditis elegans GN=CELE_F40F8.5 PE=1 SV=1	tr|H2L2A1|H2L2A1_CAEEL	3	4
Uncharacterized protein F08B12.4 OS=Caenorhabditis elegans GN=F08B12.4 PE=4 SV=2	sp|Q19191|YZ10_CAEEL (+1)	3	1
Uncharacterized protein OS=Caenorhabditis elegans GN=CELE_F09D12.2 PE=4 SV=1	tr|O44472|O44472_CAEEL	3	0
Copine family protein 2 OS=Caenorhabditis elegans GN=cpna-2 PE=2 SV=4	sp|Q09221|CPNA2_CAEEL	3	0
Uncharacterized protein OS=Caenorhabditis elegans GN=CELE_T02B11.8 PE=4 SV=1	tr|Q1XFZ1|Q1XFZ1_CAEEL	3	0
60S acidic ribosomal protein P2 OS=Caenorhabditis elegans GN=rpa-2 PE=3 SV=2	sp|O01504|RLA2_CAEEL	4	18
Acyl-CoA-binding protein homolog 1 OS=Caenorhabditis elegans GN=acbp-1 PE=3 SV=1	sp|O01805|ACBP1_CAEEL	4	11
Transcription factor BTF3 homolog OS=Caenorhabditis elegans GN=icd-1 PE=1 SV=1	sp|Q18885|BTF3_CAEEL	4	11
ATPase inhibitor mai-2, mitochondrial OS=Caenorhabditis elegans GN=mai-2 PE=3 SV=1	sp|O44441|ATIF2_CAEEL	4	9
DAF-16/FOXO Controlled, germline Tumor affecting OS=Caenorhabditis elegans GN=dct-16 PE=1 SV=1	tr|Q9XX57|Q9XX57_CAEEL	4	8

Uncharacterized protein OS=Caenorhabditis elegans GN=CELE_K03E5.2 PE=1 SV=2	tr|O61848|O61848_CAEEL	4	5
Protein unc-87 OS=Caenorhabditis elegans GN=unc-87 PE=1 SV=3	sp|P37806|UNC87_CAEEL (+1)	5	30
Troponin T OS=Caenorhabditis elegans GN=mup-2 PE=2 SV=1	sp|Q27371|TNNT_CAEEL	5	26
Uncharacterized protein C53C9.2 OS=Caenorhabditis elegans GN=C53C9.2 PE=3 SV=2	sp|Q09936|YSE2_CAEEL	5	6
TropoNin T OS=Caenorhabditis elegans GN=tnt-2 PE=1 SV=1	tr|Q7Z072|Q7Z072_CAEEL	6	29
Uncharacterized protein OS=Caenorhabditis elegans GN=C06A8.3 PE=1 SV=1	tr|Q17698|Q17698_CAEEL	6	19
40S ribosomal protein S12 OS=Caenorhabditis elegans GN=rps-12 PE=3 SV=2	sp|P49196|RS12_CAEEL	6	14
Prion-like-(Q/N-rich)-domain-bearing protein OS=Caenorhabditis elegans GN=pqn-59 PE=1 SV=2	tr|O61708|O61708_CAEEL	6	3
Myosin regulatory light chain 2 OS=Caenorhabditis elegans GN=mlc-2 PE=3 SV=1	sp|P19626|MLR2_CAEEL	6	1
ATP synthase subunit OS=Caenorhabditis elegans GN=atp-5 PE=1 SV=2	tr|Q17763|Q17763_CAEEL	7	20
ATP synthase subunit OS=Caenorhabditis elegans GN=atp-4 PE=1 SV=1	tr|O16517|O16517_CAEEL	8	27
40S ribosomal protein S21 OS=Caenorhabditis elegans GN=rps-21 PE=1 SV=1	sp|P49197|RS21_CAEEL	8	13
Plant Late Embryo Abundant (LEA) related OS=Caenorhabditis elegans GN=lea-1 PE=1 SV=1	tr|G5EEF0|G5EEF0_CAEEL (+7)	10	0
Uncharacterized protein OS=Caenorhabditis elegans GN=lev-11 PE=1 SV=1	tr|A0A0K3ARB5|A0A0K3ARB5_CAEEL	15	38
ProGRaNulin homolog OS=Caenorhabditis elegans GN=pgrn-1 PE=1 SV=1	tr|Q9U362|Q9U362_CAEEL	19	16
Myosin, essential light chain OS=Caenorhabditis elegans GN=mlc-3 PE=3 SV=1	sp|P53014|MLE_CAEEL (+1)	22	35
CaLponIn-liKe proteins OS=Caenorhabditis elegans GN=clik-1 PE=1 SV=1	tr|Q23050|Q23050_CAEEL	26	49
Tropomyosin isoforms a/b/d/f OS=Caenorhabditis elegans GN=lev-11 PE=1 SV=1	sp|Q22866|TPM1_CAEEL	36	76

Supplementary Table 4. Gene ontology (GO) enrichment analysis of proteins co-immunoprecipitated with α-synuclein from *C. elegans* lysates. The biological processes are ranked by fold enrichment within the GO over-representation analysis of biological processes using PANTHER "GO biological process complete" (pantherdb.org/geneListAnalysis.do). The *C. elegans* genome was used as the reference list. Only biological processes with a false discovery rate (FDR) ≤0.05 are listed.

Biological Process	Gene Ontology Term	# of Genes from Input	# of Genes Expected	Fold Enrichment
'De novo' protein folding	(GO:0006458)	4	0.09	45.17
Chaperone cofactor-dependent protein refolding	(GO:0051085)	3	0.08	35.87
'De novo' posttranslational protein folding	(GO:0051084)	3	0.08	35.87
Protein folding	(GO:0006457)	6	0.46	13.11
ATP metabolic process	(GO:0046034)	6	0.52	11.62
Translation	(GO:0006412)	9	1.29	6.98
Peptide biosynthetic process	(GO:0043043)	9	1.31	6.85
Peptide metabolic process	(GO:0006518)	10	1.57	6.35
Cellular protein-containing complex assembly	(GO:0034622)	9	1.45	6.22
Amide biosynthetic process	(GO:0043604)	9	1.46	6.18
Cytoskeleton organization	(GO:0007010)	9	1.54	5.84
Cellular amide metabolic process	(GO:0043603)	10	1.97	5.08
Protein-containing complex assembly	(GO:0065003)	9	1.79	5.04
Organelle organization	(GO:0006996)	17	5.39	3.15
Cellular component organization or biogenesis	(GO:0071840)	24	8.43	2.85
Cellular component organization	(GO:0016043)	22	7.83	2.81

Supplementary Table 5. Known entities are compounds previously shown to reduce α-synuclein oligomers in cell or animal models in hypothesis-driven studies (see references). The corresponding number of abstracts of published abstracts identified and used by IBM Watson for Drug Discovery Predictive Analytics for each drug on the date of ranking is provided.

Drug Name	# of Abstracts
17-AAG[21,48,49,61,65]	816
AICAR[56]	446
Anle183b[59]	5
Baicalein[57]	1000
Curcumin[58]	1000
Cyclosporine[49]	1000
Geldanamycin[54]	1000
Isorhynchophylline[60]	51
Myricetin[58]	1000
PD169316[49]	176
Resveratrol[56]	1687
Rosmarinic acid[58]	1021
SB239063[49]	122
Tacrolimus[55]	1000
TBBz[49]	19

Supplementary Table 6. *In silico* ranking of compounds by IBM Watson for Drug Discovery Predictive Analytics based on their likelihood of inhibiting α-synuclein aggregation. Candidate entities (N=620) are oral medications currently prescribed for treatment of various human diseases and listed in the Ontario Drug Benefit Program formulary (formulary.health.gov.on.ca/formulary/). Known entities (N=15) are in bold. Ranking is shown for each of the known entities when removed from the known set and added to the candidate set during leave-one-out validation analysis. Compounds highlighted in blue were selected for testing *in vitro*.

Drug Name	IBM Watson Ranking	Score (arbitrary)
**Curcumin**		**0.026572896**
**Resveratrol**		**0.024891593**
Verapamil	1	0.02391447
Tretinoin	2	0.023665065
Caffeine	3	0.02212228
Dexamethasone	4	0.021293124
**PD169316**		**0.020369345**
**SB239063**		**0.020314941**
Sirolimus	5	0.019989299
Indomethacin	6	0.019799477
Etoposide	7	0.019230978
**Baicalein**		**0.018408176**
**Geldanamycin**		**0.018190702**
Estradiol	8	0.018186088
Celecoxib	9	0.017885722
Framycetin	10	0.016386577
**17-AAG**		**0.013898701**
**Isorhynchophylline**		**0.01359914**
**TBBz**		**0.011656517**
**Myricetin**		**0.011613904**
**Rosmarinic acid**		**0.011587948**
Sulindac	11	0.01155053
Tamoxifen	12	0.01073828
**Cyclosporine**		**0.010528345**
Piroxicam	13	0.010327113
Minocycline	14	0.01029561
Ketoconazole	15	0.010211005
Alitretinoin	16	0.009433155
**AICAR**		**0.009317443**
Lovastatin	17	0.009266705
Erlotinib	18	0.009241609
Ascorbic acid	19	0.009162541
Flurbiprofen	20	0.008901568
Tetracycline	21	0.00810332

Naproxen	22	0.007928475
Propranolol	23	0.007212216
Temozolomide	24	0.006998555
Sennosides	25	0.006921414
Imatinib	26	0.006875301
Allopurinol	27	0.006825175
Galantamine	28	0.00679403
Rifabutin	29	0.006793076
Calcitriol	30	0.006743945
Vincristine	31	0.006730485
Pioglitazone	32	0.00670246
Mercaptopurine	33	0.006681412
Melphalan	34	0.006625598
Losartan	35	0.006591429
Ibuprofen	36	0.006536924
Theophylline	37	0.006147827
Acetaminophen	38	0.00607345
Hydrocortisone	39	0.005921451
Pseudoephedrine	40	0.005077118
Collagenase	41	0.004775753
Auranofin	42	0.004727561
Pentoxifylline	43	0.004670313
Miconazole	44	0.004667356
Polyethylene glycol	45	0.004591185
**Anle183b**		**0.004580211**
Ferrous sulfate	46	0.004574955
Mycophenolate	47	0.004554131
Filgrastim	48	0.004544292
Donepezil	49	0.004538387
Fenofibrate	50	0.004521217
Leflunomide	51	0.00451132
Simvastatin	52	0.004507803
Ganciclovir	53	0.004500985
Fludarabine	54	0.004491515
Memantine	55	0.004487818
Chlorambucil	56	0.004487548
Rasagiline	57	0.004482605
Selegiline	58	0.004472337
Busulfan	59	0.004456739
Valganciclovir	60	0.004424035
Ursodiol	61	0.004421451
Nifedipine	62	0.004411532
Scopolamine	63	0.004410799
Adalimumab	64	0.004402775
Cinacalcet	65	0.004400428

Fluvastatin	66	0.004397216
Infliximab	67	0.004393745
Ethinyl estradiol	68	0.004356137
Cyclophosphamide	69	0.004347513
5-aminosalicylic acid	70	0.0043463
Azathioprine	71	0.004340647
Telmisartan	72	0.004332182
Ketoprofen	73	0.004315727
Methotrexate	74	0.004306706
Acyclovir	75	0.004302158
Valacyclovir	76	0.004300905
Pimecrolimus	77	0.004245589
Prednisolone	78	0.004164329
Prednisone	79	0.004105229
Methylprednisolone	80	0.004085007
Fluorouracil	81	0.002607249
Clotrimazole	82	0.002584098
Tryptophan	83	0.002567123
Ferrous fumarate	84	0.002503152
Dantrolene	85	0.00250279
Polystyrene sodium sulfonate	86	0.002495491
Nimodipine	87	0.002493982
Trifluoperazine	88	0.002492626
Thioguanine	89	0.002487549
Bicalutamide	90	0.00246725
Glucagon	91	0.002465363
Nicotinic acid	92	0.002461031
Carbachol	93	0.00244903
Daunorubicin	94	0.002444007
Mebendazole	95	0.002439156
Riluzole	96	0.002413749
Pyridoxine	97	0.002399361
Sodium aurothiomalate	98	0.00238003
Polyvinylpyrrolidone	99	0.00237589
Bezafibrate	100	0.002367541
Clodronate	101	0.002355484
Atropine	102	0.002354346
Methoxsalen	103	0.002343206
Diltiazem	104	0.002334782
Digoxin	105	0.00232269
Dipyridamole	106	0.00232206
Dextromethorphan	107	0.002320006
Rivastigmine	108	0.002317793

Procarbazine	109	0.002315562
Hydroxyurea	110	0.002312519
Amantadine	111	0.002303632
Sulfasalazine	112	0.002296055
Metformin	113	0.00228144
Etanercept	114	0.002281027
Penicillamine	115	0.002280191
Doxycycline	116	0.002279374
Fusidic acid	117	0.002278715
Candesartan	118	0.002277497
Carbidopa	119	0.00227743
Clobetasol	120	0.002272162
Imiquimod	121	0.002272057
Pamidronate	122	0.002268646
Probenecid	123	0.002267309
Benserazide	124	0.002266379
Pramipexole	125	0.002266219
Trihexyphenidyl	126	0.00226031
**Tacrolimus**		**0.002258231**
Acitretin	127	0.002254541
Levodopa	128	0.002248402
Betamethasone	129	0.00224665
Secukinumab	130	0.002234528
Pilocarpine	131	0.002228631
Hydroxychloroquine	132	0.002228131
Olsalazine	133	0.00222565
Calcipotriol	134	0.002216495
Interferon alfa-2b	135	0.002206356
Codeine	136	0.002201571
Ustekinumab	137	0.00219197
Pancrelipase	138	0.00219166
Epoetin alfa	139	0.002170286
Epinephrine	140	0.002165059
Amphotericin b	141	0.002147183
Metoclopramide	142	0.00212225
Clonazepam	143	0.002122124
Diazepam	144	0.002083002
Acetylsalicylic acid	145	4.36E-04
Clofibrate	146	4.23E-04
Zinc	147	4.06E-04
Methimazole	148	3.82E-04
Methylcellulose	149	3.78E-04
Isotretinoin	150	3.56E-04
Sodium citrate	151	3.54E-04
Trifluridine	152	3.51E-04

Mefenamic acid	153	3.47E-04
Meloxicam	154	3.42E-04
Thiamine	155	3.28E-04
Nystatin	156	3.26E-04
Gramicidin	157	3.25E-04
Etodolac	158	3.21E-04
Cholecalciferol	159	3.20E-04
Citric acid	160	3.20E-04
Mineral oil	161	3.17E-04
Insulin	162	3.12E-04
Calcitonin	163	3.07E-04
Calcium	164	3.06E-04
Propylthiouracil	165	3.05E-04
Tiaprofenic acid	166	3.04E-04
Piperonyl butoxide	167	3.01E-04
Amiloride	168	2.98E-04
Lanreotide	169	2.90E-04
Atenolol	170	2.84E-04
Sulfinpyrazone	171	2.83E-04
Oxaprozin	172	2.83E-04
Conjugated estrogens	173	2.80E-04
Diclofenac	174	2.80E-04
Folic acid	175	2.80E-04
Lomustine	176	2.69E-04
Glyburide	177	2.64E-04
Atovaquone	178	2.62E-04
Metoprolol	179	2.60E-04
Estropipate	180	2.60E-04
Permethrin	181	2.59E-04
Altretamine	182	2.55E-04
Flumethasone	183	2.53E-04
Pyrethrins	184	2.51E-04
Leucovorin	185	2.50E-04
Etidronate	186	2.50E-04
Progesterone	187	2.48E-04
Bethanechol	188	2.48E-04
Alendronate	189	2.46E-04
Nabumetone	190	2.43E-04
Octreotide	191	2.42E-04
Desmopressin	192	2.41E-04
Azelaic acid	193	2.38E-04
Ergocalciferol	194	2.36E-04
Flutamide	195	2.35E-04
Trimebutine	196	2.34E-04

Zoledronic acid	197	2.32E-04
Capecitabine	198	2.30E-04
Diphenhydramine	199	2.30E-04
Aluminum hydroxide	200	2.29E-04
Sodium biphosphate	201	2.29E-04
Chloroquine	202	2.28E-04
Anagrelide	203	2.26E-04
Benzydamine	204	2.26E-04
Flunarizine	205	2.26E-04
Potassium chloride	206	2.24E-04
Prazosin	207	2.24E-04
Nilutamide	208	2.23E-04
Estramustine	209	2.21E-04
Cyanocobalamin	210	2.20E-04
Polyvinyl alcohol	211	2.20E-04
Dextran 70	212	2.17E-04
Benzoyl peroxide	213	2.15E-04
Lodoxamide	214	2.11E-04
Captopril	215	2.09E-04
Iodine	216	2.07E-04
Alfacalcidol	217	2.07E-04
Cimetidine	218	2.03E-04
Cosyntropin	219	2.00E-04
Megestrol	220	1.99E-04
Isosorbide	221	1.97E-04
Ferrous gluconate	222	1.97E-04
Methazolamide	223	1.94E-04
Thyroid	224	1.92E-04
Risedronate	225	1.91E-04
Docusate	226	1.89E-04
Guaifenesin	227	1.88E-04
Isopropyl myristate	228	1.88E-04
Misoprostol	229	1.86E-04
Omeprazole	230	1.85E-04
Petrolatum	231	1.84E-04
Acetazolamide	232	1.82E-04
Exemestane	233	1.81E-04
Gemfibrozil	234	1.78E-04
Pyridostigmine	235	1.75E-04
Ketotifen	236	1.74E-04
Sildenafil	237	1.73E-04
Thyrotropin alpha	238	1.72E-04
Minoxidil	239	1.72E-04
Floctafenine	240	1.68E-04
Quinagolide	241	1.67E-04

Nitroglycerin	242	1.67E-04
Butalbital	243	1.67E-04
Bosentan	244	1.66E-04
Procainamide	245	1.65E-04
Phenytoin	246	1.64E-04
Testosterone	247	1.63E-04
Naphazoline	248	1.63E-04
Loperamide	249	1.62E-04
Mexiletine	250	1.62E-04
Ketorolac	251	1.62E-04
Flecainide	252	1.62E-04
Gentamicin	253	1.61E-04
Amphetamine	254	1.61E-04
Sulfamethoxazole	255	1.61E-04
Famotidine	256	1.59E-04
Ranitidine	257	1.59E-04
Sucralfate	258	1.59E-04
Cholestyramine	259	1.57E-04
Letrozole	260	1.53E-04
Trimethoprim	261	1.53E-04
Hydralazine	262	1.53E-04
Penicillin	263	1.52E-04
Brimonidine	264	1.49E-04
Bromocriptine	265	1.49E-04
Ethynodiol	266	1.49E-04
Magnesium	267	1.49E-04
Pantoprazole	268	1.48E-04
Lidocaine	269	1.48E-04
Iodochlorhydroxyquin	270	1.47E-04
Latanoprost	271	1.45E-04
Anastrozole	272	1.45E-04
Gliclazide	273	1.44E-04
Benazepril	274	1.43E-04
Lansoprazole	275	1.39E-04
Fludrocortisone	276	1.37E-04
Niacin	277	1.36E-04
Eptifibatide	278	1.36E-04
Montelukast	279	1.36E-04
Aprepitant	280	1.36E-04
Danazol	281	1.35E-04
Tramadol	282	1.34E-04
Felodipine	283	1.34E-04
Aflibercept	284	1.32E-04
Lactulose	285	1.31E-04
Ethambutol	286	1.30E-04

Ceftriaxone	287	1.30E-04
Ofloxacin	288	1.29E-04
Furosemide	289	1.29E-04
Haloperidol	290	1.29E-04
Polymyxin b	291	1.28E-04
Bisacodyl	292	1.27E-04
Terbinafine	293	1.26E-04
Diflucortolone	294	1.25E-04
Rosuvastatin	295	1.25E-04
Oxtriphylline	296	1.25E-04
Repaglinide	297	1.22E-04
Mefloquine	298	1.21E-04
Rifampin	299	1.20E-04
Triamcinolone	300	1.20E-04
Ciprofloxacin	301	1.20E-04
Cabergoline	302	1.19E-04
Clobetasone	303	1.19E-04
Meclizine	304	1.19E-04
Pravastatin	305	1.18E-04
Neostigmine	306	1.17E-04
Darifenacin	307	1.17E-04
Amcinonide	308	1.17E-04
Finasteride	309	1.17E-04
Atorvastatin	310	1.17E-04
Pyrazinamide	311	1.16E-04
Tobramycin	312	1.16E-04
Gabapentin	313	1.16E-04
Ethopropazine	314	1.15E-04
Tranylcypromine	315	1.14E-04
Raloxifene	316	1.14E-04
Flavoxate	317	1.13E-04
Ticlopidine	318	1.12E-04
Ezetimibe	319	1.11E-04
Disopyramide	320	1.11E-04
Triptorelin	321	1.10E-04
Rabeprazole	322	1.10E-04
Buserelin	323	1.10E-04
Orciprenaline	324	1.09E-04
Amiodarone	325	1.09E-04
Desonide	326	1.09E-04
Betahistine	327	1.08E-04
Goserelin	328	1.08E-04
Saquinavir	329	1.06E-04
Propafenone	330	1.06E-04
Ocriplasmin	331	1.05E-04

Nizatidine	332	1.05E-04
Terconazole	333	1.05E-04
Dimethicone	334	1.05E-04
Bacitracin	335	1.05E-04
Levonorgestrel	336	1.04E-04
Denosumab	337	1.03E-04
Fluorometholone	338	1.02E-04
Nadolol	339	1.00E-04
Zanamivir	340	1.00E-04
Desipramine	341	1.00E-04
Citalopram	342	9.98E-05
Acarbose	343	9.97E-05
Psyllium	344	9.81E-05
Granisetron	345	9.74E-05
Metronidazole	346	9.73E-05
Clopidogrel	347	9.69E-05
Nitrofurantoin	348	9.68E-05
Clonidine	349	9.63E-05
Amitriptyline	350	9.57E-05
Famciclovir	351	9.56E-05
Azithromycin	352	9.56E-05
Ampicillin	353	9.55E-05
Cefuroxime	354	9.55E-05
Sotalol	355	9.53E-05
Oxycodone	356	9.51E-05
Sodium cromoglycate	357	9.47E-05
Proguanil	358	9.42E-05
Sumatriptan	359	9.22E-05
Fluconazole	360	9.21E-05
Morphine	361	9.21E-05
Ondansetron	362	9.16E-05
Povidone	363	9.12E-05
Leuprolide	364	9.10E-05
Triamterene	365	9.07E-05
Norgestimate	366	9.05E-05
Colesevelam	367	9.05E-05
Fluocinonide	368	9.00E-05
Drospirenone	369	8.99E-05
Acebutolol	370	8.92E-05
Betaxolol	371	8.90E-05
Rifaximin	372	8.89E-05
Sulfadiazine	373	8.86E-05
Colestipol	374	8.72E-05
Norethindrone	375	8.71E-05
Oseltamivir	376	8.71E-05

Sodium phosphate	377	8.60E-05
Medroxyprogesterone	378	8.59E-05
Sodium picosulfate	379	8.56E-05
Dienogest	380	8.55E-05
Cyproterone	381	8.54E-05
Prasugrel	382.5	8.48E-05
Ticagrelor	382.5	8.48E-05
Ulipristal	384	8.45E-05
Midazolam	385	8.37E-05
Tinzaparin	386	8.27E-05
Valsartan	387	8.20E-05
Irbesartan	388	8.20E-05
Ranibizumab	389	8.12E-05
Sodium fusidate	390	8.11E-05
Glimepiride	391	7.93E-05
Clozapine	392	7.90E-05
Diphenoxylate	393	7.88E-05
Baclofen	394	7.87E-05
Degarelix	395	7.86E-05
Cephalexin	396	7.77E-05
Orphenadrine	397	7.76E-05
Beclomethasone	398	7.73E-05
Fluoxetine	399	7.70E-05
Hydrocodone	400	7.46E-05
Moxifloxacin	401	7.41E-05
Brinzolamide	402	7.35E-05
Norfloxacin	403	7.31E-05
Botulinum toxin type a	404	7.27E-05
Zolmitriptan	405	7.21E-05
Voriconazole	406	7.13E-05
Zidovudine	407	6.94E-05
Dextroamphetamine	408	6.92E-05
Linezolid	409	6.92E-05
Naloxone	410	6.91E-05
Doxylamine	411	6.90E-05
Didanosine	412	6.88E-05
Sitagliptin	413	6.83E-05
Linagliptin	414	6.82E-05
Tranexamic acid	415	6.82E-05
Warfarin	416	6.75E-05
Cloxacillin	417	6.75E-05
Fentanyl	418	6.70E-05
Fondaparinux	419	6.64E-05
Aripiprazole	420	6.62E-05

Darbepoetin alfa	421	6.62E-05
Budesonide	422	6.61E-05
Desogestrel	423	6.55E-05
Spironolactone	424	6.44E-05
Erythromycin	425	6.37E-05
Methyldopa	426	6.35E-05
Tizanidine	427	6.34E-05
Lorazepam	428	6.32E-05
Terbutaline	429	6.29E-05
Dalteparin	430	6.25E-05
Entacapone	431	6.20E-05
Rivaroxaban	432	6.15E-05
Meperidine	433	6.12E-05
Clindamycin	434	6.10E-05
Polyquad	435	6.07E-05
Carvedilol	436	6.06E-05
Rizatriptan	437	6.05E-05
Hydromorphone	438	6.04E-05
Lithium	439	5.99E-05
Naratriptan	440	5.99E-05
Piperacillin	441	5.85E-05
Clavulanic acid	442	5.85E-05
Doxazosin	443	5.83E-05
Lisinopril	444	5.78E-05
Pimozide	445	5.77E-05
Empagliflozin	446	5.73E-05
Ropinirole	447	5.61E-05
Almotriptan	448	5.49E-05
Procyclidine	449	5.49E-05
Loratadine	450	5.48E-05
Promethazine	451	5.45E-05
Domperidone	452	5.44E-05
Levobunolol	453	5.39E-05
Tigecycline	454	5.35E-05
Fosfomycin	455	5.35E-05
Mupirocin	456	5.33E-05
Salbutamol	457	5.30E-05
Cefepime	458	5.30E-05
Cyclobenzaprine	459	5.15E-05
Hydroxyzine	460	5.05E-05
Apixaban	461	5.01E-05
Dorzolamide	462	4.92E-05
Nortriptyline	463	4.91E-05
Perampanel	464	4.89E-05
Risperidone	465	4.88E-05

Olanzapine	466	4.87E-05
Primidone	467	4.77E-05
Canagliflozin	468	4.73E-05
Amoxicillin	469	4.71E-05
Hydrochlorothiazide	470	4.71E-05
Clarithromycin	471	4.71E-05
Oxazepam	472	4.70E-05
Fluticasone	473	4.69E-05
Dimenhydrinate	474	4.67E-05
Adefovir	475.5	4.64E-05
Lamivudine	475.5	4.64E-05
Ritonavir	477	4.63E-05
Amlodipine	478	4.60E-05
Bisoprolol	479	4.58E-05
Midodrine	480	4.52E-05
Buprenorphine	481	4.52E-05
Ipratropium	482	4.50E-05
Benztropine	483	4.49E-05
Methadone	484	4.47E-05
Dabigatran	485	4.33E-05
Saxagliptin	486	4.32E-05
Enalapril	487	4.29E-05
Frovatriptan	488	4.29E-05
Eletriptan	489	4.22E-05
Tadalafil	490	4.19E-05
Varenicline	491	4.12E-05
Bimatoprost	492	4.03E-05
Tetrabenazine	493	4.02E-05
Temazepam	494	3.97E-05
Tamsulosin	495	3.92E-05
Phenelzine	496	3.82E-05
Flunisolide	497	3.79E-05
Metolazone	498	3.78E-05
Mometasone	499	3.75E-05
Cetirizine	500	3.74E-05
Tenofovir	501	3.72E-05
Eplerenone	502	3.67E-05
Entecavir	503	3.65E-05
Quinapril	504	3.62E-05
Olmesartan	505	3.62E-05
Indapamide	506	3.61E-05
Chlorpromazine	507	3.60E-05
Ramipril	508	3.60E-05
Perindopril	509	3.60E-05
Cilazapril	510	3.57E-05

Nelfinavir	511	3.57E-05
Fosinopril	512	3.57E-05
Eprosartan	513	3.57E-05
Topiramate	514	3.56E-05
Formoterol	515	3.51E-05
Labetalol	516	3.48E-05
Timolol	517	3.46E-05
Olodaterol	518	3.45E-05
Alfuzosin	519	3.45E-05
Pindolol	520	3.45E-05
Glycopyrronium	521	3.44E-05
Trazodone	522	3.43E-05
Ethosuximide	523	3.37E-05
Quetiapine	524	3.36E-05
Escitalopram	525	3.28E-05
Chlorthalidone	526	3.21E-05
Valproate	527	3.05E-05
Buspirone	528	3.03E-05
Cefoxitin	529	3.00E-05
Fluphenazine	530	2.98E-05
Tazobactam	531	2.97E-05
Cefixime	532	2.92E-05
Zopiclone	533	2.90E-05
Terazosin	534	2.81E-05
Silodosin	535	2.79E-05
Mirabegron	536	2.72E-05
Umeclidinium	537	2.72E-05
Pizotyline	538	2.64E-05
Delavirdine	539	2.53E-05
Oxybutynin	540	2.50E-05
Imipramine	541	2.49E-05
Carbamazepine	542	2.44E-05
Paroxetine	543	2.40E-05
Mirtazapine	544	2.39E-05
Flupentixol	545	2.39E-05
Nabilone	546	2.37E-05
Lamotrigine	547	2.36E-05
Moclobemide	548	2.32E-05
Alprazolam	549	2.30E-05
Vigabatrin	550	2.29E-05
Stavudine	551	2.24E-05
Dutasteride	552	2.13E-05
Pregabalin	553	2.07E-05
Cefadroxil	554	1.98E-05
Travoprost	555	1.97E-05

Perphenazine	556	1.94E-05
Trospium	557	1.90E-05
Salmeterol	558	1.88E-05
Tiotropium	559	1.86E-05
Lacosamide	560	1.84E-05
Sertraline	561	1.84E-05
Efavirenz	562	1.82E-05
Methsuximide	563	1.78E-05
Vilanterol	564	1.77E-05
Levetiracetam	565	1.76E-05
Triazolam	566	1.75E-05
Clorazepate	567	1.72E-05
Nitrazepam	568	1.71E-05
Bromazepam	569	1.70E-05
Indacaterol	570	1.68E-05
Prochlorperazine	571	1.66E-05
Tolterodine	572	1.45E-05
Naltrexone	573	1.43E-05
Chlordiazepoxide	574	1.38E-05
Ciclesonide	575	1.35E-05
Clomipramine	576	1.33E-05
Modafinil	577	1.31E-05
Zolpidem	578	1.28E-05
Flurazepam	579	1.27E-05
Lurasidone	580	1.25E-05
Oxcarbazepine	581	1.22E-05
Clobazam	582	1.19E-05
Abacavir	583	1.17E-05
Olopatadine	584	1.11E-05
Cefazolin	585	1.11E-05
Pericyazine	586	9.02E-06
Bupropion	587	8.73E-06
Methotrimeprazine	588	8.61E-06
Fluvoxamine	589	8.40E-06
Methylphenidate	590	7.96E-06
Thiothixene	591	7.81E-06
Lisdexamfetamine	592	7.59E-06
Trimipramine	593	7.46E-06
Eslicarbazepine	594	7.24E-06
Doxepin	595	6.19E-06
Cefaclor	596	5.52E-06
Cefprozil	597	4.23E-06
Divalproex	598	3.59E-06
Solifenacin	599	3.56E-06
Duloxetine	600	3.39E-06

Venlafaxine	601	2.98E-06
Atazanavir	602	2.90E-06
Fesoterodine	603	2.77E-06
Atomoxetine	604	2.69E-06
Loxapine	605	2.59E-06
Ziprasidone	606	2.43E-06
Asenapine	607	2.41E-06
Paliperidone	608	2.19E-06
Lopinavir	609	1.97E-06
Emtricitabine	610	1.95E-06
Nevirapine	611	1.67E-06
Darunavir	612	1.62E-06
Cobicistat	616.5	1.39E-06
Dolutegravir	616.5	1.39E-06
Elvitegravir	616.5	1.39E-06
Etravirine	616.5	1.39E-06
Fosamprenavir	616.5	1.39E-06
Maraviroc	616.5	1.39E-06
Raltegravir	616.5	1.39E-06
Rilpivirine	616.5	1.39E-06

Supplementary Table 7. Forty compounds ranked highly by an AI-driven *in silico* platform based on its predictions of their ability to reduce α-synuclein oligomers were tested *in vitro*.

Ranking	Drug Name	Canonical Drug Function/Indication	Potential Links to α-synuclein and PD
1	Verapamil	Voltage-dependent calcium channel blocker, various cardiac diseases	Calcium channel blockers within its family are associated with reduced PD risk[1]
2	Tretinoin	Acne medication, acute promyelocytic leukemia	Unknown
3	Caffeine	Inhibitor of adenosine A2A receptor, commonly used stimulant	Experimentally rescues rodent models of PD,[2] associated with reduced PD risk[3]
4	Dexamethasone	Corticosteroid, anti-inflammatory	Induces anti-inflammatory effect and reduces toxic α-synuclein aggregates[4]
5	Rapamycin	Immunosuppressant, post-transplantation	Induces autophagy to reduce α-synuclein aggregates in numerous models of PD[5]
6	Indomethacin	NSAID, COX inhibitor	Exhibits neuroprotective effect in mouse models of PD, similar to other anti-inflammatory drugs[6]
7	Etoposide	DNA topoisomerase inhibitor, anti-cancer	Unknown
8	Estradiol	Female sex hormone, prescribed for hormone replacement therapy	Exhibits neuroprotective effect on dopaminergic neurons exposed to MPP+ *in vitro*[7]
9	Celecoxib	NSAID, COX2 inhibitor, anti-arthritic	Rescues dopaminergic dysfunction in a rodent LPS-induced neurotoxicity model[8]
11	Sulindac	NSAID, COX inhibitor	Unknown
12	Tamoxifen	Estrogen receptor inhibitor, anti-breast cancer	Unknown
13	Piroxicam	NSAID, non-selective COX inhibitor	Exhibited anti-fibrillogenic activity in a drug screen of various NSAIDs[9]
14	Minocycline	Broad spectrum tetracycline-class antibiotic	Anti-inflammatory in MPTP mouse model[10]
15	Ketoconazole	Topical antimycotic	Rescued neurodegeneration in *Drosophila* model of α-synuclein expression[11]
16	Alitretinoin	Eczema topical ointment	Unknown
17	Lovastatin	Inhibitor of de novo cholesterol synthesis, lowers blood cholesterol levels	Reduces α-synuclein abundance in mouse models[12]
18	Erlotinib	EGFR inhibitor, chemotherapy	Unknown
19	Ascorbic acid	Common micronutrient, provides antioxidant function	Exhibits neuroprotective effect in *Drosophila* neurodegeneration[13]
20	Flurbiprofen	NSAID, possible COX inhibitor	Exhibited anti-fibrillogenic activity in a drug screen of various NSAIDs[14]

21	Tetracycline	Broad-spectrum antibiotic	Unknown
22	Naproxen	NSAID, nonselective COX inhibitor	Exhibited weak anti-fibrillogenic activity in a drug screen of various NSAIDs[14]
23	Propranolol	Anti-hypertensive drug, beta-adrenergic receptor blocker	Increases abundance of *SNCA* transcript[15]
24	Temozolomide	Alkylating agent, chemotherapy	Unknown
26	Imatinib	Bcr-Abl inhibitor, chemotherapy	Derivatives of this compound rescue neurodegeneration in an MPTP mouse model[16]
27	Allopurinol	Uric acid synthesis inhibitor, gout	Unknown
28	Galantamine	Acetylcholinesterase inhibitor, Alzheimer's disease	Clinically used in PD dementia[17]
29	Rifabutin	Antibiotic, tuberculosis, and *Mycobacterium avium complex*	Unknown
30	Calcitriol	Increases blood calcium levels	Related compounds shown to reduce α-synuclein aggregation[18]
31	Vincristine	Microtubule destabilizing agent	Unknown
32	Pioglitazone	PPAR-γ inducer, diabetes	Neuroprotective in rodent models of PD[19,20]
33	Mercaptopurine	Purine analog, chemotherapy	Putative activator of neuroprotective factors[21]
34	Melphalan	DNA alkylating agent, chemotherapy	Unknown
35	Losartan	Angiotensin receptor II antagonist, hypertension	Exhibits neuroprotective effect in cell and MPTP rodent model[22,23]
36	Ibuprofen	NSAID, COX inhibitor	Clinical correlation with reduced PD risk[24]
37	Theophylline	Bronchodilator	Unknown
38	Acetaminophen	Analgesic, anti-pyretic	Rescues *C. elegans* models of dopaminergic neurodegeneration[25]
39	Hydrocortisone	Steroid, anti-inflammatory	Unknown
42	Auranofin	Anti-inflammatory, arthritis	Exhibits neuroprotective effect by reducing inflammation[26]
43	Pentoxifylline	Nonselective phosphodiesterase inhibitor	Rescues 6-OHDA rat model of PD[27]
46	Iron sulfate	Iron nutrient supplement	Shows interaction with α-synuclein, possibly inducing sequestration[28]

These highly ranked compounds were excluded from *in vitro* testing for the following reasons: framycetin (ranking 10) and miconazole (ranking 44) are topical medications (not oral medications), sennosides (ranking 25) and polyethylene glycol (ranking 45) are laxatives, pseudoephedrine (ranking 40) is a controlled substance with limited access, and collagenase (ranking 41) is an enzyme (not a small molecule).

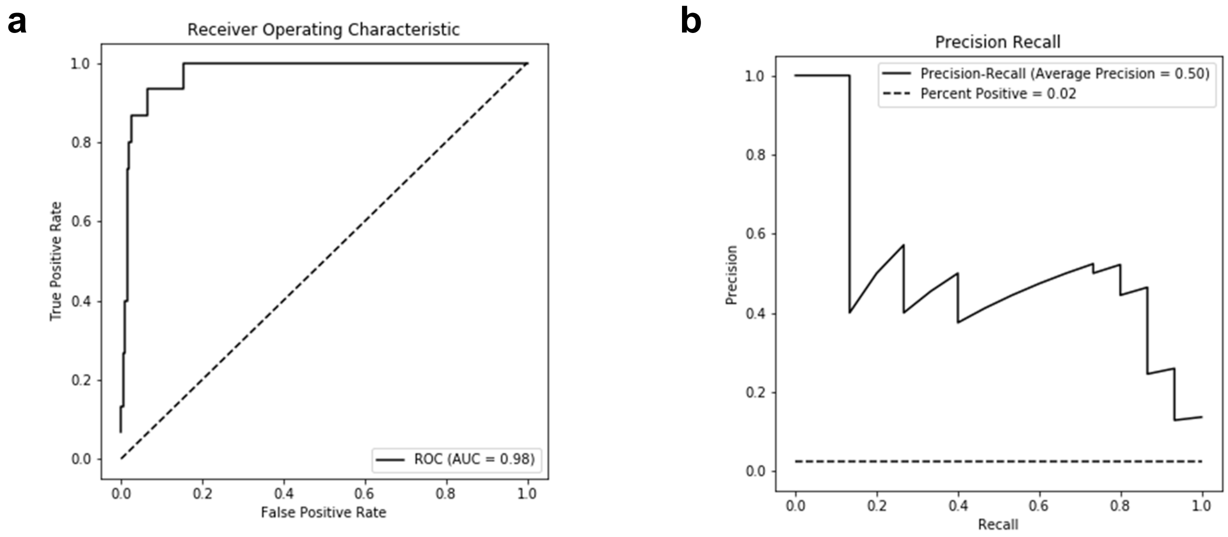

Supplementary Figure 1. Performance of the algorithm used to rank candidate drugs by similarity to the known entities that reduce α-synuclein oligomers. (a) Receiver-operating characteristic (ROC) curve. The semantic framework was assessed using leave-one-out validation: one known entity was placed among the other candidate drugs and ranked, repeating for each individual drug in the known set. Individual points were connected using a stepwise curve. The area under curve (AUC) value is 0.98. A dashed line, with an AUC score of 0.5, represents a null hypothesis curve where the rankings of known entities made by IBM Watson for Drug Discovery Predictive Analytics would be completely random. (b) Precision-recall curve. The curve has an average precision of 0.50 and is always above the dashed line, a precision of 0.02 (the percent of positives), which represents a model with no skill.

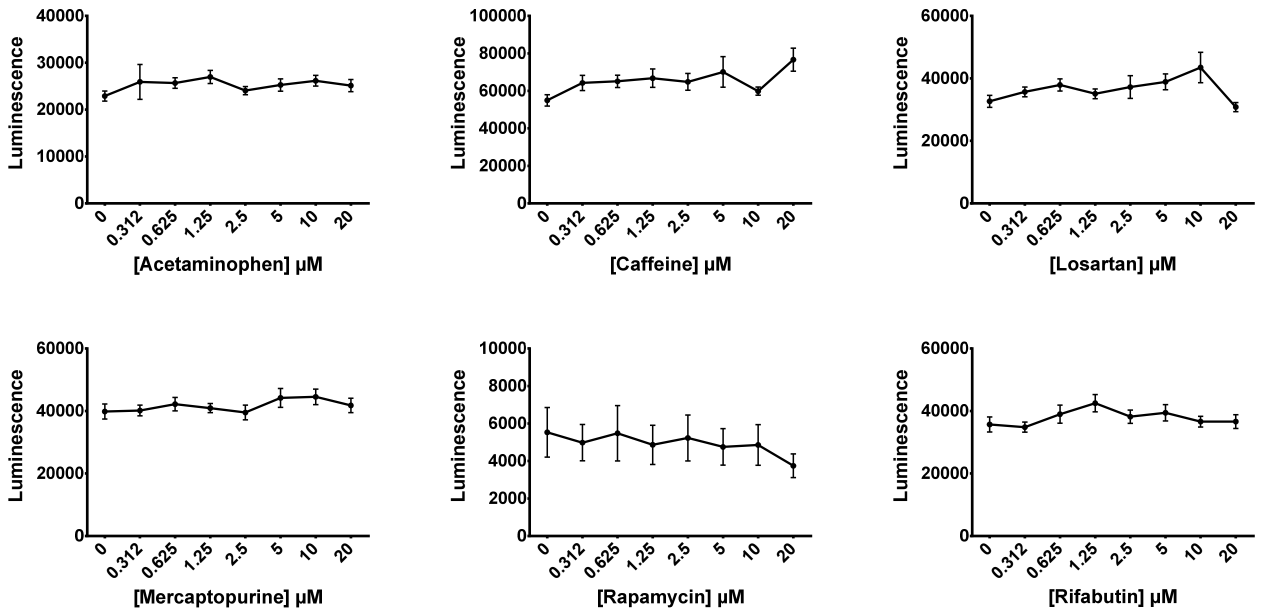

Supplementary Figure 2. Acetaminophen, caffeine, losartan, mercaptopurine, rapamycin, and rifabutin do not alter the activity of full-length *Gaussia* luciferase alone. Raw luminescence data (measured in relative luciferase units) corresponding with normalized data in Figure 5c are shown here (N=2 with n=8 replicates for each concentration).

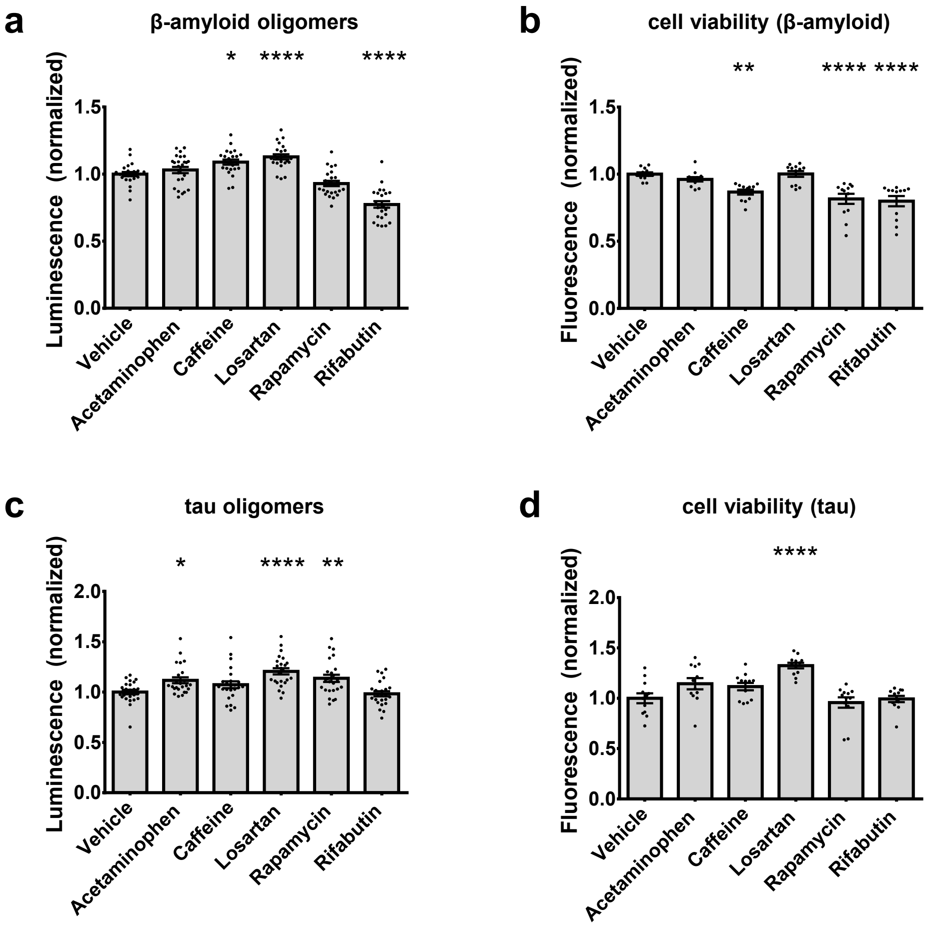

Supplementary Figure 3. Effects of acetaminophen, caffeine, losartan, mercaptopurine, rapamycin, and rifabutin on β-amyloid and tau oligomers *in vitro*. Testing of compounds found to reduce α-synuclein oligomers for their effects on (a) β-amyloid oligomer levels *in vitro* using a *Gaussia* luciferase protein-fragment complementation cell assay (N=3 with n=8 replicates for each treatment) and (b) viability in these cells (N=3 with n=4 replicates for each treatment). Testing of compounds found to reduce α-synuclein oligomers for their effects on (c) tau oligomer levels *in vitro* using a *Gaussia* luciferase protein-fragment complementation cell assay (N=3 with n=8 replicates for each treatment) and (d) viability in these cells (N=3 with n=4 replicates for each treatment) (two-tailed t-test, *$p<0.05$, **$p<0.01$, ****$p<0.0001$). Each data point represents an individual replicate.

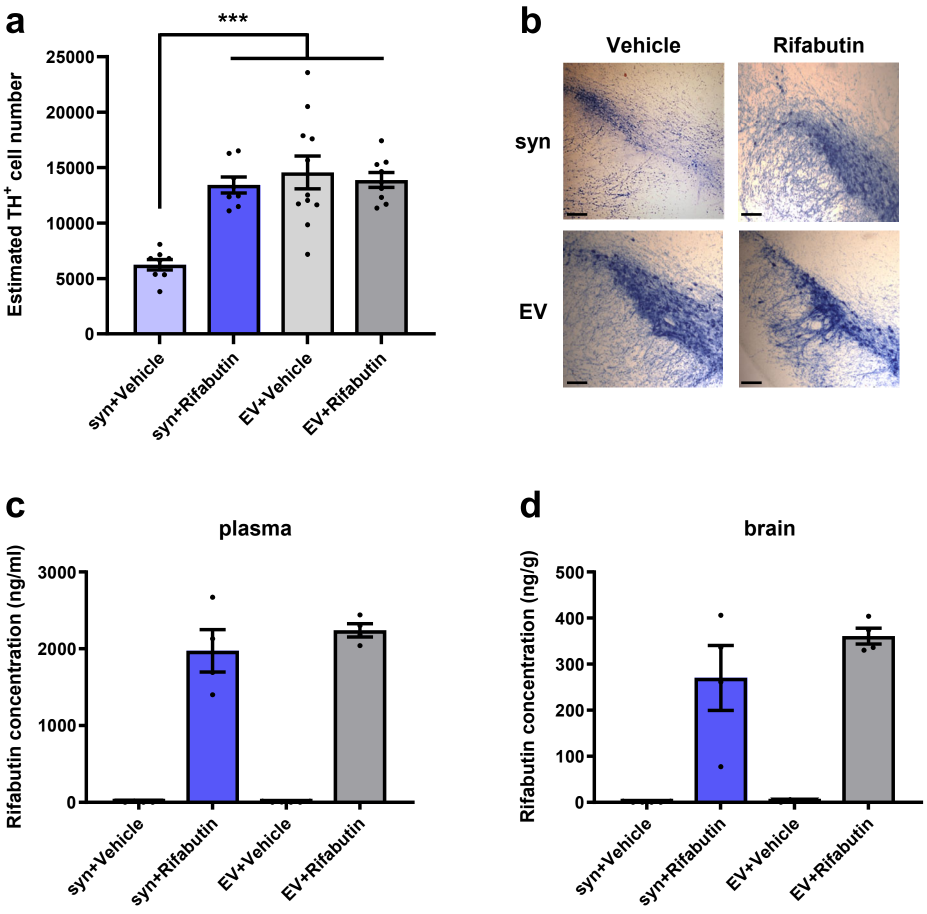

Supplementary Figure 4. Rifabutin penetrates the blood-brain barrier and reduces nigral dopaminergic neuron loss in an AAV-based α-synuclein rat model. (a) Dopaminergic neuron loss in the SN was induced by stereotactic injection of human A53T α-synuclein-expressing AAV (syn). AAV lacking the A53T α-synuclein open reading frame was used as an empty vector control (EV). Animals injected with AAV-A53T α-synuclein and treated with vehicle exhibited a substantial loss of dopaminergic (TH+) cells. Rifabutin rescued α-synuclein-mediated dopaminergic neurodegeneration in the SN, restoring TH+ cell numbers to those comparable to animals injected with AAV-EV and treated with vehicle or rifabutin (n=8-11 animals per treatment group, one-way ANOVA with Tukey's post-hoc test, ***p< 0.001). (b) Representative images of TH staining of the SN injected with AAV-A53T α-synuclein or AAV-EV from rats treated with vehicle or rifabutin. Scale bars are 200 µm. Steady-state rifabutin concentrations in (c) plasma and (d) saline-perfused brain tissue from a subset of animals (n=4 per treatment group). Mean brain-to-plasma drug concentration ratio (± SD) for rifabutin treated animals was 0.15 ± 0.04.

References

1. Pasternak, B. *et al.* Use of calcium channel blockers and Parkinson's disease. *Am. J. Epidemiol.* **175**, 627–635 (2012).
2. Ascherio, A. *et al.* Prospective study of caffeine consumption and risk of Parkinson's disease in men and women. *Ann. Neurol.* **50**, 56–63 (2001).
3. Chen, J. F. *et al.* Neuroprotection by caffeine and A(2A) adenosine receptor inactivation in a model of Parkinson's disease. *J. Neurosci.* **21**, RC143 (2001).
4. McLeary, F. A. *et al.* Dexamethasone inhibits copper-induced alpha-synuclein aggregation by a metallothionein-dependent mechanism. *Neurotox. Res.* **33**, 229–238 (2018).
5. Decressac, M. *et al.* TFEB-mediated autophagy rescues midbrain dopamine neurons from α-synuclein toxicity. *Proc. Natl. Acad. Sci.* **110**, E1817–E1826 (2013).
6. La Vitola, P. *et al.* Alpha-synuclein oligomers impair memory through glial cell activation and via Toll-like receptor 2. *Brain. Behav. Immun.* **69**, 591–602 (2018).
7. Sawada, H. *et al.* Estradiol protects dopaminergic neurons in a MPP+ Parkinson's disease model. *Neuropharmacology* **42**, 1056–1064 (2002).
8. Kaizaki, A. *et al.* Celecoxib reduces brain dopaminergic neuronal dysfunction, and improves sensorimotor behavioral performance in neonatal rats exposed to systemic lipopolysaccharide. *J. Neuroinflammation* **10**, 45 (2013).
9. Khansari, P. S. & Coyne, L. NSAIDs in the treatment and/or prevention of neurological disorders. *Inflammopharmacology* **20**, 159–167 (2012).
10. Du, Y. *et al.* Minocycline prevents nigrostriatal dopaminergic neurodegeneration in the MPTP model of Parkinson's disease. *Proc. Natl. Acad. Sci.* **98**, 14669–14674 (2001).

11. Styczyńska-Soczka, K., Zechini, L. & Zografos, L. Validating the predicted effect of astemizole and ketoconazole using a Drosophila model of Parkinson's disease. *Assay Drug Dev. Technol.* **15**, 106–112 (2017).

12. Koob, A. O. *et al.* Lovastatin ameliorates alpha-synuclein accumulation and oxidation in transgenic mouse models of alpha-synucleinopathies. *Exp. Neurol.* **221**, 267–274 (2010).

13. Khan, S. *et al.* Effect of L-ascorbic Acid on the climbing ability and protein levels in the brain of Drosophila model of Parkinson's disease. *Int. J. Neurosci.* **122**, 704–709 (2012).

14. Hirohata, M., Ono, K., Morinaga, A. & Yamada, M. Non-steroidal anti-inflammatory drugs have potent anti-fibrillogenic and fibril-destabilizing effects for alpha-synuclein fibrils in vitro. *Neuropharmacology* **54**, 620–627 (2008).

15. Mittal, S. *et al.* β2-Adrenoreceptor is a regulator of the α-synuclein gene driving risk of Parkinson's disease. *Science* **357**, 891–898 (2017).

16. Hebron, M. L., Lonskaya, I. & Moussa, C. E.-H. Nilotinib reverses loss of dopamine neurons and improves motor behavior via autophagic degradation of α-synuclein in Parkinson's disease models. *Hum. Mol. Genet.* **22**, 3315–3328 (2013).

17. Emre, M. Treatment of dementia associated with Parkinson's disease. *Parkinsonism Relat. Disord.* **13**, S457–S461 (2007).

18. Rcom-H'cheo-Gauthier, A. N., Meedeniya, A. C. B. & Pountney, D. L. Calcipotriol inhibits α-synuclein aggregation in SH-SY5Y neuroblastoma cells by a Calbindin-D28k-dependent mechanism. *J. Neurochem.* **141**, 263–274 (2017).

19. Yu, H. C., Feng, S. F., Chao, P. L. & Lin, A. M. Y. Anti-inflammatory effects of pioglitazone on iron-induced oxidative injury in the nigrostriatal dopaminergic system. *Neuropathol. Appl. Neurobiol.* **36**, 612–622 (2010).

20. Ulusoy, G. K. *et al.* Effects of pioglitazone and retinoic acid in a rotenone model of Parkinson's disease. *Brain Res. Bull.* **85**, 380–384 (2011).

21. Kim, C.-H., Leblanc, P. & Kim, K.-S. 4-amino-7-chloroquinoline derivatives for treating Parkinson's disease: implications for drug discovery. *Expert Opin. Drug Discov.* **11**, 337–341 (2016).

22. Grammatopoulos, T. N., Outeiro, T. F., Hyman, B. T. & Standaert, D. G. Angiotensin II protects against α-synuclein toxicity and reduces protein aggregation in vitro. *Biochem. Biophys. Res. Commun.* **363**, 846–851 (2007).

23. Grammatopoulos, T. N. *et al.* Angiotensin type 1 receptor antagonist losartan, reduces MPTP-induced degeneration of dopaminergic neurons in substantia nigra. *Mol. Neurodegener.* **2**, 1 (2007).

24. Gagne, J. J. & Power, M. C. Anti-inflammatory drugs and risk of Parkinson disease. *Neurology* **74**, 995–1002 (2010).

25. Locke, C. J., Fox, S. A., Caldwell, G. A. & Caldwell, K. A. Acetaminophen attenuates dopamine neuron degeneration in animal models of Parkinson's disease. *Neurosci. Lett.* **439**, 129–133 (2008).

26. Madeira, J. M. *et al.* Novel protective properties of auranofin: inhibition of human astrocyte cytotoxic secretions and direct neuroprotection. *Life Sci.* **92**, 1072–1080 (2013).

27. Neves, K. R. T. *et al.* Pentoxifylline neuroprotective effects are possibly related to its anti-inflammatory and TNF-alpha inhibitory properties, in the 6-OHDA model of Parkinson's disease. *Parkinsons Dis.* **2015,** 108179 (2015).

28. Davies, P., Moualla, D. & Brown, D. R. Alpha-synuclein is a cellular ferrireductase. *PLOS ONE* **6**, e15814 (2011).
